# QTL for Main Stem Node Number and Its Response to Plant Densities in 144 Soybean FW-RILs

**DOI:** 10.3389/fpls.2021.666796

**Published:** 2021-08-20

**Authors:** Wen-Xia Li, Ping Wang, Hengxing Zhao, Xu Sun, Tao Yang, Haoran Li, Yongqin Hou, Cuiqiao Liu, Mahfishan Siyal, Rameez Raja veesar, Bo Hu, Hailong Ning

**Affiliations:** ^1^Key Laboratory of Soybean Biology, Ministry of Education, Key Laboratory of Soybean Biology and Breeding/Genetics, Ministry of Agriculture, Northeast Agricultural University, Harbin, China; ^2^High Education Institute, Huaiyin Institute of Technology, Huai'an, China

**Keywords:** soybean, node number of main stem, response to density, linkage analysis and GWAS, gene mining

## Abstract

Although the main stem node number of soybean [*Glycine max* (L.) Merr. ] is an important yield-related trait, there have been limited studies on the effect of plant density on the identification of quantitative trait loci (QTL) for main stem node number (MSNN). To address this issue, here, 144 four-way recombinant inbred lines (FW-RILs) derived from Kenfeng 14, Kenfeng 15, Heinong 48, and Kenfeng 19 were used to identify QTL for MSNN with densities of 2.2 × 10^5^ (D1) and 3 × 10^5^ (D2) plants/ha in five environments by linkage and association studies. As a result, the linkage and association studies identified 40 and 28 QTL in D1 and D2, respectively, indicating the difference in QTL in various densities. Among these QTL, five were common in the two densities; 36 were singly identified for response to density; 12 were repeatedly identified by both response to density and phenotype of two densities. Thirty-one were repeatedly detected across various methods, densities, and environments in the linkage and association studies. Among the 24 common QTL in the linkage and association studies, 15 explained a phenotypic variation of more than 10%. Finally, *Glyma.06G094400, Glyma.06G147600, Glyma.19G160800.1*, and *Glyma.19G161100* were predicted to be associated with MSNN. These findings will help to elucidate the genetic basis of MSNN and improve molecular assistant selection in high-yield soybean breeding.

## Introduction

Since soybean was one of the important crops worldwide, it has been an ongoing aim for soybean breeders to breed high yield cultivars in order to meet increasing global demand. As a major plant architecture trait, main stem node number (MSNN) affects soybean seed yield (Yao et al., 2015; Chang et al., 2018), for it is related with seed yield characters, such as logging, number of pods per plant, and days to flowering (Chapman et al., 2003; Zhang et al., 2004; Egli, 2013). MSNN is a typical quantitative trait, and the interaction of the genotype and the environment complicates the study on genetic basis. Therefore, molecular markers are widely used to locate quantitative trait loci (QTL) to reveal the molecular mechanism of MSNN in soybean yield. To date, Soybase (https://www.soybase.org/) has listed 37 QTL for MSNN by genetic linkage analysis (Zhang et al., 2004; Chen et al., 2007; Gai et al., 2007; Li et al., 2010; Liu et al., 2011; Moongkanna et al., 2011; Yao et al., 2015) and 11 quantitative trait nucleotides (QTNs) by genome-wide association study (GWAS) (Fang et al., 2017).

Genetic linkage analysis is an effective and traditional method to identify genetic intervals that associated plant phenotypes of traits (Tanksley et al., 1992). With the further development of DNA chip technology, single nucleotide polymorphism (SNP) has been widely used in high-density genetic linkage map construction and mapping of QTL (Hyten et al., 2010; Kim et al., 2010; Akond et al., 2013; Jun et al., 2014; Lee et al., 2015). GWAS can identify QTNs in genome regions based on the density of SNP, with advantages of high detection accuracy, high throughput, low cost, and time-saving. However, high false positive ratio is its inevitable defect. More researchers supported the viewpoint that the combination of linkage and association analysis was more accurate and effective than single methods (Ott et al., 2011; Liu et al., 2018; Fang et al., 2020; Zhang Y. C. et al., 2020). However, identifying the location of MSNN QTL for soybean using the combination of linkage analysis and GWAS analysis has not yet been discussed.

Population selection is the most important foundation for the linkage and GWAS analyses to map QTL. Linkage analysis typically is based on populations derived from two parents, and its detection power is usually relatively lower because of its less genetic diversity (Zhang S. et al., 2017). GWAS analysis generally uses natural populations or germplasm resources, and the population structure problem reduces the accuracy of the results. In order to solve the problems, scientists suggested constructing a special population, such as multi-parent advanced generation inter-cross (MAGIC) (Kover et al., 2009). The great opportunity for recombination in multiple parent populations increases mapping accuracy. Abundant genetic variation improves the efficiency of the detection of QTL, and clear kinship in progenies solves the population structure problem (Cavanagh et al., 2008). Kover et al. (2009) first constructed a MAGIC population with 19 *Arabidopsis thaliana* parents, and proved that the MAGIC population had great advantages in the location of QTL by mapping several known QTL with high precision. Huang et al. (2012) created a wheat MAGIC population from four excellent Australian varieties and identified QTL for plant height and hectoliter weight successfully. Butrón et al. (2019) also identified QTL for resistance to Fusarium ear rot in a MAGIC maize population.

In this study, in order to identify more accurate QTL and further perform gene mining precisely, an FW-RIL derived from a four-way cross was used to identify QTL for the MSNN of two densities in five environments by the combination of linkage analysis and GWAS. This research will enrich MSNN QTL and improve the precision of gene mining, as well as reveal the molecular mechanisms of MSNN in response to density, which will subsequently lay the foundation for marker-assisted selection breeding to increase soybean yield.

## Materials and Methods

### Plant Materials

To construct a four-way recombinant inbred line population, four soybean varieties with different node numbers in the main stem, Kenfeng 14, Kenfeng 15, Heinong 48, and Kenfeng 19, were used as parents. In 2008, two single crosses of Kenfeng 14 × Kenfeng 15 and Heinong 48 × Kenfeng 19 were obtained in Harbin, Heilongjiang province, China, and the F_1_ was crossed as (Kenfeng 14 × Kenfeng 15) × (Heinong 48 × Kenfeng 19) in 2009. From 2010 to 2014, the progeny was self-crossed following the single seed descent method in Harbin and Yacheng, Hainan province, China. Finally, an FW-RIL population with 144 homozygous individuals was obtained and used for genetic map construction and mapping of QTL.

### Field Experiment and Trait Measurement

The field experiment was conducted in Harbin (E126.63°, N45.75°) in 2015 (E1), Keshan (E125.64°, N48.25°) in 2015 (E2), Acheng (E127.63°, N45.82°) in 2016 (E3), Shuangcheng (E126.92°, E45.75°) in 2016 (E4), and Harbin in 2016 (E5). The parents and FW-RILs were planted in a three-row 5 × 0.7 m plot in a split block design of three replications. The main block arranged the plant densities, namely, 2.2 × 10^5^ plants/ha (D1) and 3 × 10^5^ plants/ha (D2). The sub-blocks were planted lines. The management procedures followed the normal production practices.

Five mature plants of the four parents and 144 four-way recombinant inbred lines (FW-RILs) were selected randomly in the middle of each row to measure MSNN before the harvest in the field for each replication. MSNN indicated the number of nodes from the cotyledonary node to the top of the main stem. The average of the three replications was used for phenotypic data analysis.

### Genotyping and SNP Map Construction

Juvenile leaves were frozen in liquid nitrogen from the parents and FW-RIL plants, and then were ground into powder. Total genomic DNA was extracted with the CTAB method (Doyle et al., 1990) and eluted in 50-μl deionized water. SNP genotyping was conducted with SoySNP660K BeadChip at Beijing Boao Biotechnology Co. Ltd. A total of 109,676 SNPs were selected from 600,010 across 20 chromosomes, with minor allele frequency (MAF) > 0.05 and maximum SNP deletion locus <20% as criteria for the screening of SNP quality, and heterozygous loci were marked as missing to better estimate marker effect. Then, the locus was selected at each 100 kb interval along each chromosome from 3′-bottom to 5′-bottom. 2,292 high-quality SNPs on 20 chromosomes following Mendelian segregating ratio was applied to construct linkage map by the software GAPL V1.0 (Zhang S. et al., 2017). The length of the 20 linkage groups ranged from 76.4 to 329.7 cm, and the total length was 3,539.7 cm. The markers in each linkage group ranged from 16 to 316, with an average interval distance of 4.09 cm (ranging from 1.92 to 10.93 cm).

### Statistical Analysis

#### Phenotypic Variation Analysis

The maximum, minimum, and standard deviations, skewness, and kurtosis of MSNN were calculated for each density in each environment. ANOVA was conducted with SAS V 9.2. ANOVA for single environment was carried out according to the following equation:

$$\begin{array}{l} x_{ijr}=\mu +R_{r}+D_{j}+RD_{jr}+G_{i}+GD_{ij}+\;\varepsilon_{ijr} \end{array}$$

where *x*_*ijr*_is the *r*th observation of the *i*th genotype under the *j*th density in an environment; μ is the grand mean; *R*_*r*_ is the effect of main block *r*; *D*_*j*_ is the effect of density *j*; *RD*_*jr*_ is error of main block; *G*_*i*_ is the effect of genotype *i*; *GD*_*ij*_ is the interaction effect of genotype *i* by density *j*; and ε_*ijr*_ is the residual error, ε_*ijr*_ ~ *N*(0, σ^2^).

For multiple environments, joint ANOVA was conducted according to the following equation:

$$\begin{array}{l} x_{eijr}=\mu +E_{e}+E_{e}\left(R_{r}\right)+D_{j}+ED_{ej}+E_{e}\left(RD_{rj}\right)+G_{i}+GD_{ij}\\ \;+GE_{ei}+GDE_{eij}+\;\varepsilon_{eijr} \end{array}$$

where *x*_*eijr*_ is the *r*th observation of the *i*th genotype under the *j*th density in *e*th environment; μ is the grand mean; *E*_*e*_ is effect of *e*th environment; *E*_*e*_ (*R*_*r*_) is the effect of *r*th main block in *e*th environment; *D*_*j*_ is the effect of density *j*; *ED*_*ej*_ is the interaction effect of density *j* by environment *e*; *E*_*e*_ (*RD*_*rj*_) is error of main block in *e*th environment; *G*_*i*_ is the effect of genotype *i*; *GD*_*ij*_ is the interaction effect of genotype *i* by density *j*; *GE*_*ei*_ is the interaction effect of genotype *i* by environment *e*; *GDE*_*eij*_ is the interaction effect of genotype *i* by density *j* by environment *e*; and ε_*eijr*_ is the residual error, ε*e*_*ijr*_ ~ *N*(0, σ^2^).

Genotype variance, genotype × density interaction variance, and error variance were estimated *via* a mixed model. The heritability (*h*^2^) for single environment was calculated with the following equation:

$$\begin{array}{l} h^{2}=\frac{\sigma_{G}^{2}}{\sigma_{G}^{2}+\sigma_{GD}^{2}/d+\sigma^{2}/dr} \end{array}$$

The heritability (*h*^2^) for multiple environments was calculated with the following equation:

$$\begin{array}{l} h^{2}=\frac{\sigma_{G}^{2}}{\sigma_{G}^{2}+\sigma_{GD}^{2}/d+\sigma_{GDE}^{2}/de+\sigma^{2}/edr} \end{array}$$

where *h*^2^ is heritability; $\sigma_{G}^{2}$ is the variance of genotype; $\sigma_{GD}^{2}$ is the variance of genotype × density interaction; $\sigma_{GDE}^{2}$ is the variance of genotype × density × environment interaction; σ^2^ is the variance of error; *e* is the number of environments; *d* is the number of planting density; and *r* is the number of repetitions.

### Response to Density Estimation

Response to density refers to the difference in node number in response to change in density (from D1 to D2). Response to density (RD) could be evaluated according to the conditional variable method (Zhu, 1995) with the following equation:

$$\begin{array}{l} R_{D}=x_{D_{2}}-C_{D_{1}D_{2}}\left(x_{D_{1}}-{\bar{x}}_{D_{1}}\right)/V_{D_{1}} \end{array}$$

where *R*_*D*_ is the response to density; *x*_*D*1_ is the phenotype value under the density of D1;*x*_*D*2_ is the phenotype value under the density of D2; *C*_*D*1*D*2_ is the covariance between phenotypes of MSNN under the two densities; and ${\bar{x}}_{D1}$ and *V*_*D*1_ are the average and variance of MSNN under the density of D1, respectively.

### Linkage Analysis

Based on the SNP linkage map constructed above, interval mapping (IM) and inclusive composite interval mapping (ICIM) methods were used to map the QTL for MSNN in every density and environment through the PLQ function of GAPL software V1.0 (Zhang S. et al., 2017). In order to determine the existence of QTL, the scanning step was set to 1 cm, and the likelihood of odds (LOD) threshold was set to 3. The QTL were named *qlNN*-chromosome-sequence number or *qlRDNN*-chromosome-sequence number. The QTL mapped to the same marker region were given the same sequence number. QTL mapping results were mapped on chromosomes with MapChart2.1 (https://www.wur.nl/en). For QTL for MSNN detected in one interval, QTL by density effect in each environment, i.e., the additive effect over two densities, and additive × density interaction effect, were estimated. The formulas are shown as follows:

$$\begin{array}{l} \mu_{..}=\frac{1}{gdr}\underset{i,j,k}{\sum }y_{ijk}\\ \mu_{ij}=\frac{1}{r}\underset{ik}{\sum }y_{ijk}\\ G_{i}=\frac{1}{dr}\underset{j,k}{\sum }y_{ijk}-\mu_{..}\\ D_{j}=\frac{1}{gr}\underset{i,k}{\sum }y_{ijk}-\mu_{..}\\ {GD}_{ij}=\mu_{ij}-\mu_{..}-G_{i}-D_{j}\\ \sigma_{G}^{2}=\underset{i}{\sum }f_{i}G_{i}^{2}\\ \sigma_{D}^{2}=\frac{1}{d}\underset{j}{\sum }D_{j}^{2}\\ \sigma_{GD}^{2}=\underset{i,j}{\sum }f_{ij}{GD}_{ij}^{2}\\ \sigma_{p}^{2}=\frac{1}{gdr}\underset{i,j,k}{\sum }{\left(y_{ijk}-\mu_{..}\right)}^{2} \end{array}$$

where *y*_*ijk*_ is the *k*th phenotype of *i*th allelic genotype in *j*th environment, μ_.._ is the grand mean of all observation, μ_ij_ is the mean of *i*th allelic genotype in *j*th environment, *G*_*i*_ is the *i*th allele effect genotype of putative QTL, *D*_*j*_ is the *j*th density effect, *GD*_*ij*_ is the QTL × density interaction effect of *i*th allele genotype under *j*th density, $\sigma_{G}^{2}$ is the genetic variance, $\sigma_{E}^{2}$ is the variance of density effect, $\sigma_{GD}^{2}$ is the variance of the QTL × density interaction effect, $\sigma_{p}^{2}$ is the phenotypic variance, and *g, d*, and *r* are the numbers of allelic genotype, density, and replication. On the basis of estimated $\sigma_{G}^{2}$, $\sigma_{D}^{2}$, $\sigma_{GD}^{2}$, and $\sigma_{p}^{2}$, the phenotypic variation explanation ratio (%) of additive (*PVE*_*A*_) and additive × density interaction (*PVE*_*AD*_) effect were estimated by the following formula:

$$\begin{array}{l} {PVE}_{A}=\sigma_{G}^{2}\times 100/\sigma_{p}^{2}\\ {PVE}_{AD}=\sigma_{GE}^{2}\times 100/\sigma_{p}^{2} \end{array}$$

### Genome-Wide Association Studies

The analysis of population structure was performed with the software STRUCTURE V 2.3.4. The number of subpopulations value (K) was determined with STRUCTURE HARVESTER (http://taylor0.biology.ucla.edu/structureHarvester). Linkage disequilibrium (LD) was analyzed with TASSEL 5.0. The K value was 2, and the LD was 1.63 Mb. The procedure is described in detail in a previous study (Zhang et al., 2018). Then, the GWAS was conducted with the software mrMLM.GUI V3.0 (Zhang Y. W. et al., 2020). Five multiple locus GWAS methods, mrMLM (Wang et al., 2016), FASTmrMLM (Tamba et al., 2017), FASTmrEMMA (Wen et al., 2018), pLARmEB (Zhang J. et al., 2017), and ISIS EM-BLASSO (Tamba et al., 2017), were used to identify significant QTL that control MSNN and its response to density. The probability *P* in the first step was set at 0.01 for mrMLM, FASTmrMLM, pLARmEB, ISIS EM-BLASSO, and 0.005 for FASTmrEMMA. The critical LOD score was set at 3 to determine significant QTL. The QTL were named *qnNN*-chromosome-sequence number or *qnRDNN*-chromosome-sequence number.

### Candidate Gene Prediction

The QTL used to search candidate genes should satisfy the following conditions: (1) for QTL detected by linkage: should be detected in different densities, methods, or environments; explain the phenotypic variation more than 10%, and the interval length should be <600 kb; (2) for QTL detected by GWAS: should be detected in different densities, with more than two multiple locus GWAS methods, in multiple environments, or by co-location with QTL; and explain the phenotypic variation more than 10%. The Glyma.Wm82.a2.v1 gene model in Soybase (https://soybase.org/) was used to identify genes at the interval of each of the QTL (at the interval of 100 kb on either side, determined by the rate of LD decay). According to the Phytozome website (https://phytozome.jgi.doe.gov), genes highly expressed in the stem or shoot tip were selected among them. Then, the selected genes were put together to conduct pathway analysis on the Kyoto Encyclopedia of Genes and Genomes (KEGG) website (http://www.kegg.jp). Finally, the candidate genes were predicted through the results of pathway analysis combined with their homologous genes information on other crops and potential functions in GO number (https://www.ebi.ac.uk/QuickGO/) and the NCBI database (http://www.ncbi.nlm.nih.gov/).

## Results and Analysis

### Phenotypic Analysis

**Figure 1:**
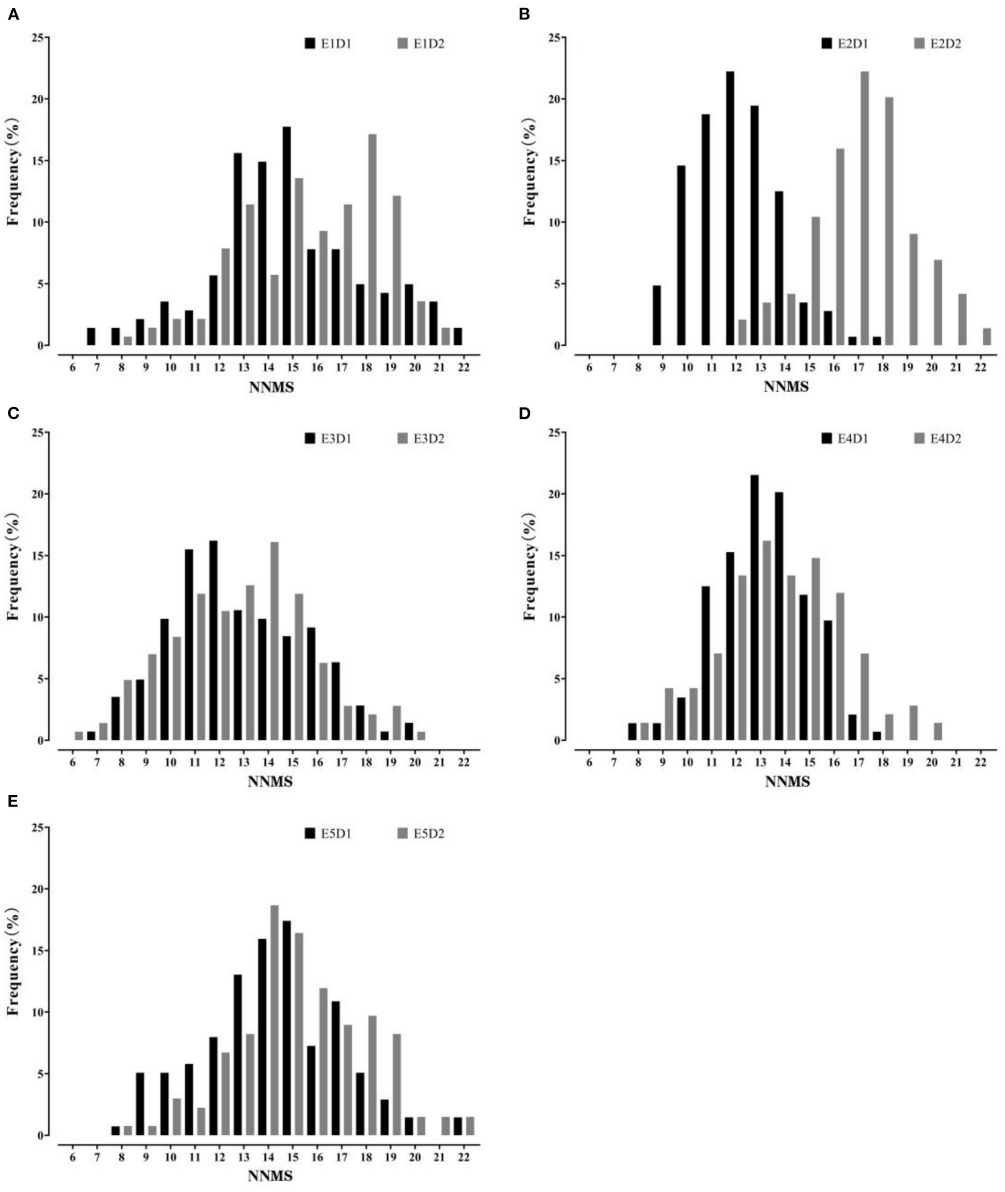
Frequency distribution of main stem node number (MSNN) in the four-way recombinant inbred line (FW-RIL) population in different treatments. **(A–E)** show the distribution in E1, E2, E3, E4, and E5, respectively; E1, Harbin in 2015; E2, Keshan in 2015; E3, Acheng in 2016; E4, Shuangcheng in 2016; E5, Harbin in 2016; D1, normal density (2.2 × 10^5^ plants/ha); D2, high density (3 × 10^5^ plants/ha).

The summary of MSNN phenotype is presented in Table 1. The data showed that the node number of the parents and FW-RIL varied with density and environment. The range of FW-RIL covered the parents, which indicated strong bilateral transgressive segregation. The skewness and kurtosis values of the FW-RIL ranged from −1 to 1, and the phenotypic data displayed a typical normal distribution (Figure 1). All the characters of phenotypic variation indicated that MSNN was controlled by large- and small-effect QTL. The significant genotypic and genotype × environment interaction variance indicated a substantial genetic variation of MSNN existed among the FW-RILs and response of genotypes to environment varied among different environments. On the basis of the significant genotype × density interaction variance and genotype × density × environment interaction variance, it was implied that the MSNN response differed to the densities and that the response varied among the environments (Table 2).

**Table 1:** Summarization of phenotype of nod number in main stem.

**Treatments^a^**	**Kenfeng 14**	**Kenfeng 15**	**Heinong 48**	**Kenfeng 19**	**FW-RIL**					
					**Min**	**Max**	**Mean**	**Std**	**Skew**	**Kurt**
E1D1	14.50	19.00	9.33	13.67	7.00	25.00	14.89	3.22	0.26	0.55
E1D2	15.00	18.50	9.70	14.20	8.00	21.00	15.74	2.81	−0.43	−0.51
E2D1	13.55	16.67	10.20	13.0	8.80	17.60	11.77	1.73	0.59	0.48
E2D2	16.80	18.50	13.00	15.00	12.00	22.00	16.68	2.04	−0.09	−0.04
E3D1	14.33	16.50	9.33	13.67	6.67	20.00	12.58	2.73	0.38	−0.38
E3D2	13.20	17.67	8.90	12.90	5.67	19.33	12.49	2.86	0.10	−0.28
E4D1	16.17	15.33	12.17	14.67	7.67	17.50	12.92	1.88	−0.21	−0.11
E4D2	15.50	16.33	12.67	13.33	8.00	20.00	13.45	2.50	0.09	−0.18
E5D1	15.00	17.00	10.50	14.50	7.50	22.50	14.02	2.80	0.12	0.15
E5D2	14.25	17.00	11.50	15.50	7.50	24.50	15.03	2.77	0.21	0.53

a *E1, Harbin in 2015; E2, Keshan in 2015; E3, Acheng in 2016; E4, Shuangcheng in 2016; E5, Harbin in 2016; D1, normal density (2.2 × 10^5^ plants/ha); D2, high density (3 × 10^5^ plants/ha)*.

**Table 2:** Variance and heritability of nod number in main stem of four-way recombinant inbred lines.

**Environment^a^**	**Genotype**		**Genotype × Density**		**Genotype × Environment**		**Genotype × Density × Environment**		**h^**2**^**
	**MS**	**Variance**	**MS**	**Variance**	**MS**	**Variance**	**MS**	**Variance**	
Joint	69.79^**^	1.75	16.13^**^	0.05	18.36^**^	0.35	15.97^**^	4.37	0.74
E1	31.83^**^	0.38	30.00^**^	8.96					0.07
E2	17.97^**^	1.47	9.13^**^	2.05					0.49
E3	32.34^**^	2.37	18.32^**^	5.04					0.44
E4	24.72^**^	2.32	10.89^**^	2.71					0.56
E5	35.81^**^	4.16	11.92^**^	3.04					0.68

a *E1, Harbin in 2015; E2, Keshan in 2015; E3, Acheng in 2016; E4, Shuangcheng in 2016; E5, Harbin in 2016*.

** *Significant difference at level of P < 0.01*.

**Figure 2:**
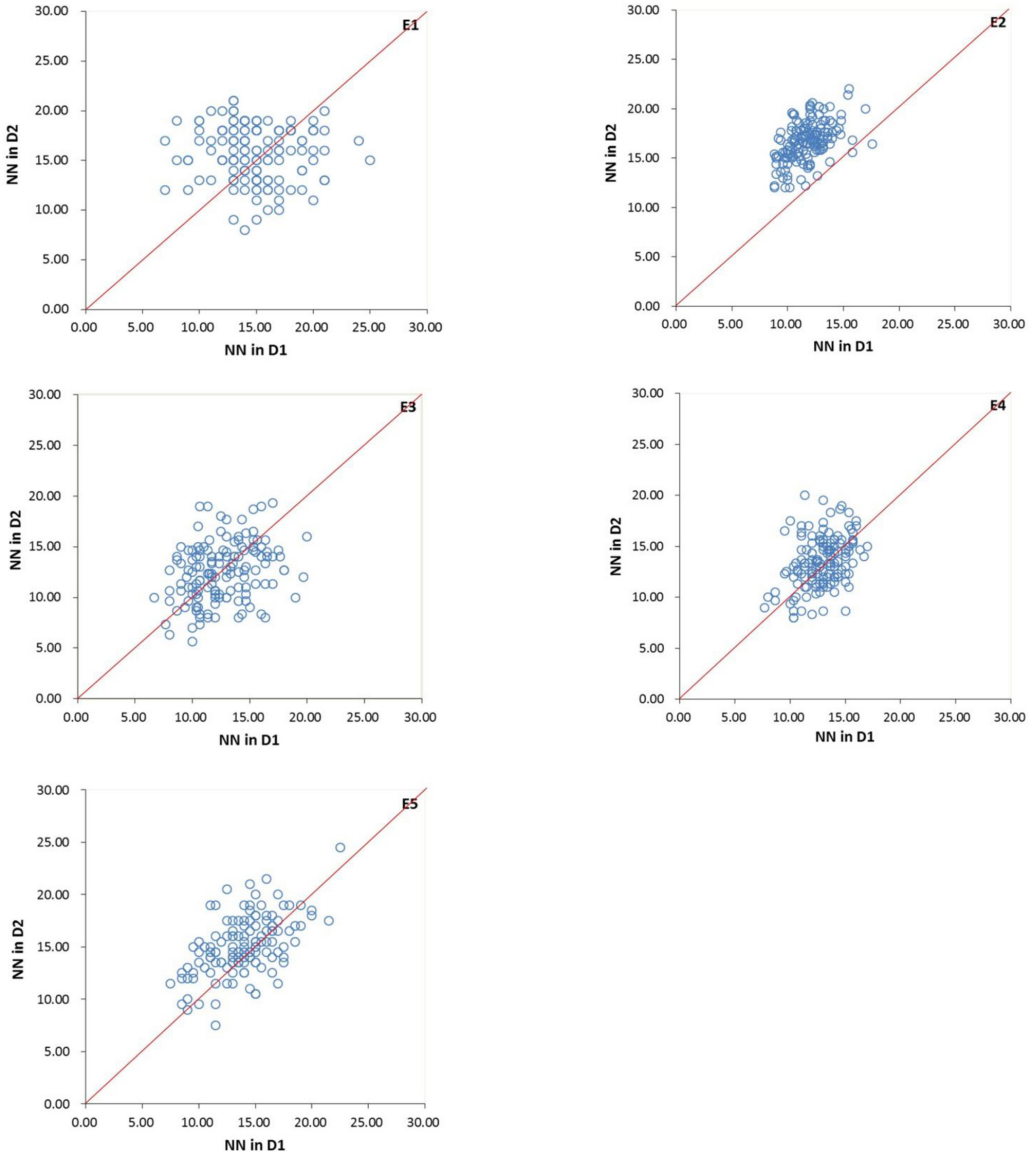
Variation in main stem node number (MSNN) under two densities in five environments for four-way recombinant inbred line (FW-RIL). E1, Harbin in 2015; E2, Keshan in 2015; E3, Acheng in 2016; E4 Shuangcheng in 2016; E5, Harbin in 2016; D1, normal density (2.2 × 10^5^ plants/ha); D2, high density (3 × 10^5^ plants/ha).

Comparing the two densities, the mean of MSNN in D2 was higher than that in D1 (Figure 2), indicating the existence of MSNN response to density. The difference in heritability among the environments suggested the genetic basis for the formation of MSNN change according to environment. The extreme difference in heritability between joint and single environments showed that the response of genotype to change in density varied among environments. The whole variation in density under environments showed it was possible to detect different QTL in various environments.

### Mapping of QTL for MSNN

**Figure 3:**
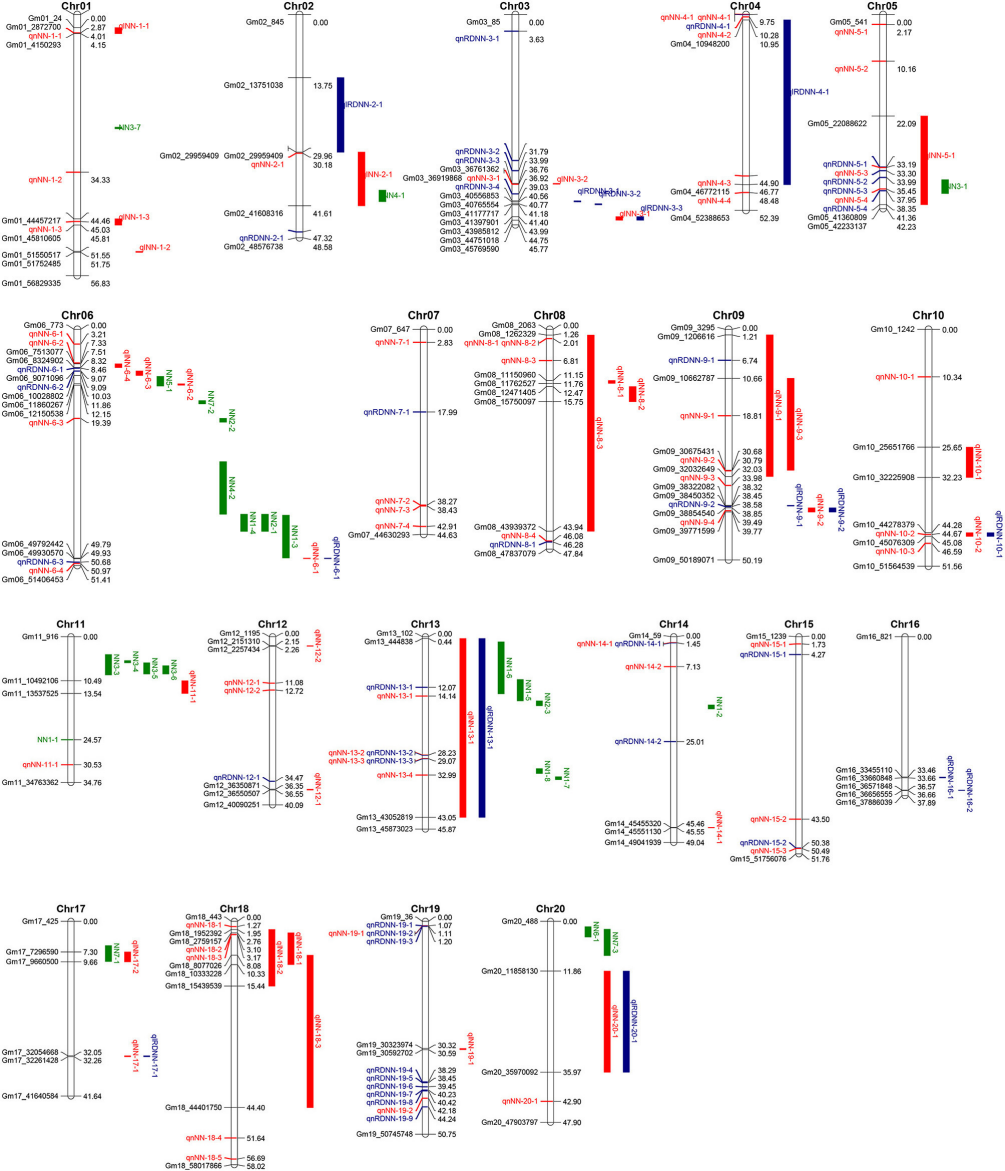
Distribution of quantitative trait loci (QTL) underlying main stem node number (MSNN) on 20 chromosomes. The red and blue colors represent QTL controlling NN and response to density change identified in present research, respectively, and the green color represents QTL underlying NN identified in previous research listed in Soybase (www.soybase.org).

**Figure 4:**
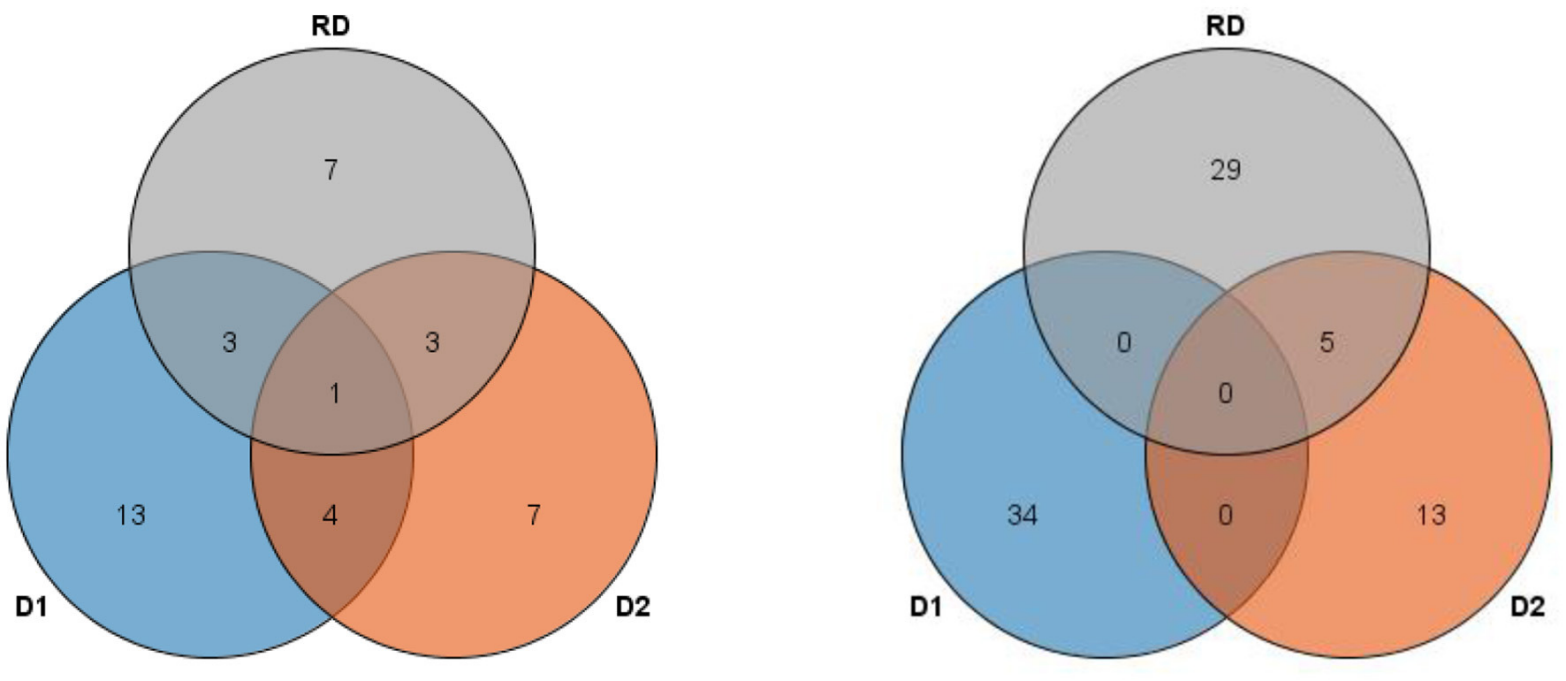
Venn figure for frequency of linkage **(left)** and genome-wide association study (GWAS) **(right)** quantitative trail loci (QTL) underlying main stem node number (MSNN) and response to density increment. D1, normal density (2.2 × 10^5^ plants/ha); D2, high density (3 × 10^5^ plants/ha); RD, response to density.

In this study, 38 QTL for MSNN were detected on 18 chromosomes (except chromosome 7 and 15) with LOD value of over 3, which explained 3.44–14.93% of phenotypic variance (Figure 3, Supplementary Table 1). Among these QTL, 13 were identified in D1, seven in D2, and five in both D1 and D2. For 14 QTL underlying the response of MSNN to density, three and three were associated singly with MSNN in D1 and D2, and one of which was associated simultaneously with MSNN in D1 and D2 (Figure 4).

**Figure 5:**
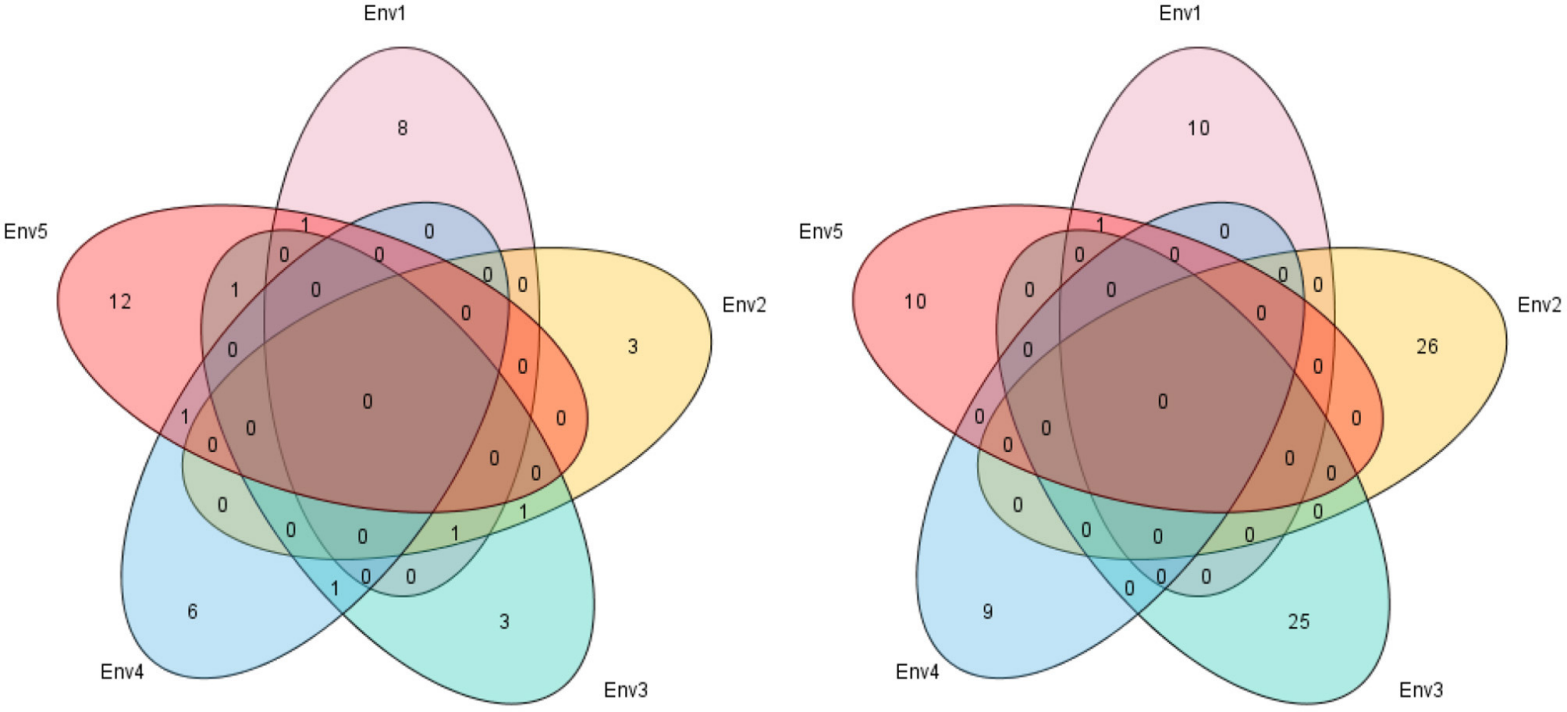
Venn figure for frequency of linkage **(left)** and genome-wide association study (GWAS) **(right)** quantitative trail loci (QTL) underlying main stem node number (MSNN) in five environments. E1, Harbin in 2015; E2, Keshan in 2015; E3, Acheng in 2016; E4, Shuangcheng in 2016; E5, Harbin in 2016.

In the five different environments, eight, three, three, six, and 12 quantitative trait loci (QTL) were detected in E1, E2, E3, E4, and E5, respectively. Six QTL could be found in more than two environments: *qlNN*-6-2 in E3 and E4; *qlNN*-8-2 in E3 and E5; *qlNN*-9-2 (*qlRDNN*-9-2) in E1, E2, and E3; *qlNN*-10-2 (*qlRDNN*-10-1) in E2 and E3; *qlNN*-13-1 (*qlRDNN*-13-1) in E4 and E5; and *qlNN*-17-1 (*qlRDNN*-17-1) in E1 and E5, respectively (Figure 5). Besides, 13 QTL could be detected with both the IM and ICIM methods, and in two densities and more than two environments, they could be considered as stable (Table 3). Among them, 12 QTL explained the phenotypic variation over 10%. *qlNN*-1-3 was detected in E2D1 with PVE of 11.01%; *qlRDNN*-3-1 was detected in E2RD with PVE of 7.57–12.07%; *qlNN*-6-1 (*qlRDNN*-6-1) was detected in E1D1, E1D2, and E1RD with PVE of 5.4–12.5%; *qlNN*-6-2 was detected in E3D1, E4D1, and E4D2 with PVE of 6.85–11.68%; *qlNN*-8-2 was detected in E3D1, E5D1, and E5D2 with PVE of 6.82–11.56%; *qlNN*-9-2 (*qlRDNN*-9-2) was detected in E1D2, E2D2, E2RD, and E3D2 with PVE of 6.63–10.50%; *qlNN*-9-3 was detected in E4D1 and E4D2 with PVE of 5.81–11.75%; *qlNN*-10-2 (*qlRDNN*-10-1) was detected in E2RD and E3D1with PVE of 6.12–11.02%; *qlNN*-13-1 (*qlRDNN*-13-1) was detected in E4RD and E5D2 with PVE of 5.16–10.07%; *qlNN*-17-1 (*qlRDNN*-17-1) was detected in E1D1 and E5RD with PVE of 3.44–12.96%; *qlNN*-18-1 was detected in E5D1 and E5D2 with PVE of 6.72–10.02%; and *qlNN*-19-1 was detected in E4D1 with PVE of 10.73–14.93%.

**Table 3:** Stable quantitative trait loci (QTL) for main stem node number (MSNN) identified under different densities in different environments with different methods.

**QTL**	**Chromosome**	**Physical position of markers (bp)**	**Length of markers (bp)**	**Treatment^a^**	**Method**	**LOD score**	**PVE^b^ (%)**	**Add1^c^**	**Add2**	**Add3**	**Add4**
***qlNN*** **-1-3**	Chr01	44,457,217…45,810,605	1,353,388	E2D1	ICIM	3.19	11.01	−0.02	0.73	0.24	−0.94
				E2D1	IM	3.19	11.01	−0.02	0.73	0.24	−0.94
***qlRDNN*** **-3-1**	Chr03	40,556,853…40,765,554	208,701	E2RD	IM	3.57	7.57	−0.51	0.57	0.57	−0.63
				E2RD	ICIM	4.60	12.07	−0.50	0.52	0.64	−0.66
*qlRDNN*-3-2	Chr03	41,177,717…41,397,901	220,184	E5RD	ICIM	3.81	9.55	−0.52	1.16	−0.95	0.31
				E5RD	IM	3.30	3.58	−0.45	1.17	−1.01	0.29
***qlNN*** **-6-1**	Chr06	49,792,442…49,930,570	138,128	E1D2	ICIM	3.28	12.50	1.52	−1.19	−0.56	0.23
**(** ***qlRDNN*** **-6-1)**				E1D1	ICIM	4.87	5.74	1.72	−0.41	−1.41	0.10
				E1D2	IM	3.41	11.72	1.69	−1.20	−0.62	0.12
				E1RD	ICIM	3.41	11.11	1.59	−1.22	−0.49	0.12
				E1RD	IM	3.16	11.11	1.70	−1.10	−0.67	0.08
				E1D1	IM	3.37	5.40	2.56	−0.77	−1.42	−0.37
***qlNN*** **-6-2**	Chr06	11,860,267…12,150,538	290,271	E4D2	ICIM	3.70	11.68	0.79	0.67	−0.08	−1.37
				E4D2	IM	3.70	8.82	0.79	0.67	−0.08	−1.37
				E3D1	ICIM	3.35	6.85	0.65	0.93	0.06	−1.64
				E4D1	ICIM	4.04	8.55	−0.23	0.77	0.41	−0.96
				E3D1	IM	3.35	6.85	0.65	0.93	0.06	−1.64
				E4D2	IM	3.80	9.13	0.31	0.86	0.50	−1.67
***qlNN*** **-8-2**	Chr08	12,471,405…15,750,097	3,278,692	E5D2	ICIM	4.40	7.14	1.37	0.60	−0.73	−1.24
				E5D1	ICIM	5.49	10.92	0.96	0.98	−1.39	−0.55
				E5D1	IM	4.72	11.56	0.88	1.23	−1.44	−0.67
				E3D1	ICIM	3.08	6.82	0.13	1.49	−0.52	−1.10
				E3D1	IM	3.08	6.82	0.13	1.49	−0.52	−1.10
***qlNN*** **-9-2**	Chr09	38,854,540…39,771,599	917,059	E1D2	ICIM	3.02	10.50	−1.09	−0.09	−0.02	1.19
**(** ***qlRDNN*** **-9-2)**				E3D2	ICIM	3.07	7.94	−0.91	0.97	−0.86	0.81
				E2D2	IM	3.61	9.91	−0.79	1.06	0.07	−0.34
				E2RD	IM	3.17	6.63	−0.76	0.84	0.12	−0.20
***qlNN*** **-9-3**	Chr09	10,662,787…30,675,431	20,012,644	E4D1	IM	3.29	5.81	0.28	−0.04	0.64	−0.88
				E4D2	ICIM	3.73	11.75	−1.92	0.49	1.03	0.39
***qlNN*** **-10-2**	Chr10	44,278,379…45,076,309	797,930	E3D1	ICIM	3.02	6.12	−0.06	0.80	−1.31	0.56
**(** ***qlRDNN*** **-10-1)**				E3D1	IM	3.02	6.12	−0.06	0.80	−1.31	0.56
				E2RD	IM	3.37	8.41	−1.05	−0.16	0.56	0.65
				E2RD	ICIM	3.72	11.02	−0.75	−0.43	0.59	0.60
***qlNN*** **-13-1**	Chr13	444,838…43,052,819	42,607,981	E4RD	ICIM	3.26	10.07	2.29	−0.62	−0.90	−0.77
**(** ***qlRDNN*** **-13-1)**				E4RD	IM	3.26	10.07	2.29	−0.62	−0.90	−0.77
				E5D2	ICIM	3.35	5.16	1.34	0.25	0.33	−1.92
				E5D2	IM	3.05	8.31	1.81	0.07	0.05	−1.93
***qlNN*** **-17-1**	Chr17	32,054,668…32,261,428	206,760	E1D1	ICIM	9.51	12.96	0.82	1.95	−1.20	−1.57
**(** ***qlRDNN*** **-17-1)**				E1D1	IM	3.12	4.28	−0.13	1.51	−0.36	−1.02
				E5RD	ICIM	3.63	9.47	−0.88	1.04	−0.59	0.44
				E5RD	IM	3.06	3.44	−0.87	1.03	−0.62	0.46
***qlNN*** **-18-1**	Chr18	2,759,157…10,333,228	7,574,071	E5D1	ICIM	4.89	10.02	1.09	−1.32	−0.67	0.90
				E5D2	IM	3.57	9.51	2.16	−1.21	−1.08	0.13
				E5D2	ICIM	3.45	6.72	2.10	−1.33	−0.65	−0.12
***qlNN*** **-19-1**	Chr19	30,323,974…30,592,702	268,728	E4D1	ICIM	4.83	14.93	−0.19	0.70	−1.18	0.67
				E4D1	IM	4.34	10.73	−0.03	0.68	−1.26	0.61

a *D1, the first (normal) density (2.2 × 10^5^ plants/has); D2, the second (high) density (3 × 10^5^ plants/ha); RD: response to density; E1, Harbin in 2015; E2, Keshan in 2015; E3, Acheng in 2016; E4, Shuangcheng in 2016; E5, Harbin in 2016*.

b *PVE, phenotypic variation explained*.

c *Add1, Add2, Add3, Add4: genetic effects from Kenfeng 14, Kenfeng 15, Heinong 48, and Kenfeng 19, respectively*.

*Bold font indicates QTL with PVE > 10%*.

Of all the QTL, the genome length of 15 QTL was <600 kb, which included six of the 13 ones: *qlRDNN*-3-1, *qlRDNN*-3-2, *qlNN*-6-1 (*qlRDNN*-6-1), *qlNN*-6-2, *qlNN*-17-1 (*qlNN*-17-1), and *qlNN*-19-1. The other stable QTL were repeatedly identified at a wide interval. In addition, all of the stable QTL with genome length of <600 kb could explain the phenotypic variation more than 10% except *qlRDNN*-3-2. Consequently, these intervals might play a critical role in mining genes to regulate MSNN.

Among all the alleles from the 13 stable QTL for MSNN, the parent Kenfeng 14 carried the positive additive effect alleles for 7 QTL, Kenfeng 15 for 11 QTL, Heinong 48 for 7 QTL, and Kenfeng 19 for 8 QTL. Four, four, one and one QTL from Kenfeng14, Kenfeng 15, Heinong 48, and Kenfeng 19 could obviously increase MSNN (additive effect > 1). Oppositely, Kenfeng 14 carried the negative additive effect alleles for 0 QTL, Kenfeng 15 for 6 QTL, and Heinong 48 and Kenfeng 19 for 10 QTL. Three, two, seven and four QTL from Kenfeng 14, Kenfeng 15, Heinong 48, and Kenfeng 19 could obviously decrease MSNN (additive effect < −1) (Table 3).

In the five environments, the total of PVE_A_ and PVE_AE_ varied extremely, ranging from 1.97 (in E2) to 45.98% (in E5). It was shown that the genetic basis of MSNN response to density varied in different environments (Table 4).

**Table 4:** Additive and additive by density effect of quantitative trait loci (QTL) under two densities in each environment.

**Environment^a^**	**QTL**	**Additive effect^b^**				**Additive by density effect**								${\text{PVE}}_{\text{A}}^{\text{c}}$ **(%)**	**PVE_**AD**_** ** (%)**
		**add1**	**add2**	**add3**	**add4**	add1*D1	add2*D1	add3*D1	add4*D1	add1*D2	add2*D2	add3*D2	add4*D2		
E1	qlNN-1-2	−0.50	0.55	−0.06	0.02	−0.48	0.80	−0.31	0.00	0.48	−0.80	0.31	0.00	1.72	3.25
	qlNN-2-1	0.15	0.16	−0.30	−0.01	0.41	−0.01	−0.23	−0.16	−0.41	0.01	0.23	0.16	0.49	0.54
	qlNN-3-2	−0.32	0.64	−0.48	0.15	−0.25	0.43	0.00	−0.18	0.25	−0.43	0.00	0.18	2.27	0.82
	qlNN-5-1	0.17	−0.13	0.16	−0.20	−0.52	0.35	−0.17	0.34	0.52	−0.35	0.17	−0.34	0.28	1.18
	qlNN-9-2	−0.44	0.28	0.53	−0.37	−0.26	−0.16	0.68	−0.26	0.26	0.16	−0.68	0.26	1.76	1.51
	qlNN-10-1	0.04	0.18	0.08	−0.30	0.28	0.19	−0.51	0.03	−0.28	−0.19	0.51	−0.03	0.36	1.06
	qlNN-11-1	0.08	0.13	0.10	−0.31	0.92	−0.08	−0.54	−0.30	−0.92	0.08	0.54	0.30	0.30	2.96
	qlNN-17-1	0.13	0.25	−0.18	−0.21	0.48	0.27	−0.28	−0.47	−0.48	−0.27	0.28	0.47	0.47	1.68
	qlNN-17-2	−0.04	0.45	0.17	−0.58	−0.01	−0.06	0.19	−0.13	0.01	0.06	−0.19	0.13	1.61	0.14
	qlNN-18-2	0.23	−0.08	−0.27	0.13	0.15	0.09	−0.16	−0.08	−0.15	−0.09	0.16	0.08	0.42	0.17
	Total													15.15	16.91
E2	qlNN-9-2	0.07	0.24	−0.19	−0.12	−0.13	−0.11	0.13	0.11	0.13	0.11	−0.13	−0.11	0.33	0.15
	qlNN-1-3	−0.01	0.23	−0.16	−0.06	−0.11	0.31	0.10	−0.31	0.11	−0.31	−0.10	0.31	0.27	0.54
	qlNN-20-1	−0.13	0.29	−0.21	0.06	0.13	0.13	0.01	−0.27	−0.13	−0.13	−0.01	0.27	0.40	0.29
	Total													1.00	0.97
E3	qlNN-6-2	−0.43	0.13	−0.34	0.64	0.01	0.09	0.29	−0.38	−0.01	−0.09	−0.29	0.38	1.94	0.66
	qlNN-8-2	0.42	−0.51	−0.20	0.29	0.05	−0.02	−0.02	0.00	−0.05	0.02	0.02	0.00	1.63	0.01
	qlNN-8-3	−0.22	−0.06	0.14	0.14	0.19	−0.17	−0.27	0.24	−0.19	0.17	0.27	−0.24	0.24	0.69
	qlNN-9-2	−0.28	0.12	0.08	0.08	0.22	−0.14	0.19	−0.27	−0.22	0.14	−0.19	0.27	0.27	0.59
	qlNN-10-2	0.50	−0.44	0.03	−0.08	−0.38	0.42	−0.63	0.59	0.38	−0.42	0.63	−0.59	1.47	3.47
	Total													5.55	5.41
E4	qlNN-5-1	0.26	−0.30	−0.26	0.30	−0.16	0.16	0.06	−0.05	0.16	−0.16	−0.06	0.05	1.65	0.25
	qlNN-6-2	0.12	−0.15	0.07	−0.03	−0.12	0.25	−0.44	0.31	0.12	−0.25	0.44	−0.31	0.22	1.93
	qlNN-6-3	0.25	0.10	−0.10	−0.24	0.11	−0.12	0.25	−0.24	−0.11	0.12	−0.25	0.24	0.67	0.78
	qlNN-9-2	0.35	−0.08	0.19	−0.46	−0.09	0.08	0.29	−0.28	0.09	−0.08	−0.29	0.28	2.27	0.98
	qlNN-9-3	−0.53	0.30	0.08	0.15	0.24	−0.18	−0.02	−0.04	−0.24	0.18	0.02	0.04	1.39	0.35
	qlNN-14-1	0.57	0.11	−0.60	−0.08	−0.20	0.25	0.00	−0.06	0.20	−0.25	0.00	0.06	3.96	0.41
	qlNN-18-3	−0.27	0.12	−0.16	0.32	0.11	−0.19	0.48	−0.41	−0.11	0.19	−0.48	0.41	1.09	1.98
	qlNN-19-1	−0.01	0.27	−0.05	−0.21	0.04	−0.11	0.22	−0.15	−0.04	0.11	−0.22	0.15	0.77	0.44
	Total													14.478	9.195
E5	qlNN-1-1	0.03	−0.39	0.19	0.18	−0.29	0.30	0.33	−0.34	0.29	−0.30	−0.33	0.34	0.82	1.27
	qlNN-3-1	−1.22	0.06	0.46	0.70	0.20	−0.44	0.17	0.07	−0.20	0.44	−0.17	−0.07	4.41	0.63
	qlNN-6-4	−0.55	0.14	−0.08	0.48	−0.24	−0.24	0.15	0.32	0.24	0.24	−0.15	−0.32	2.02	0.84
	qlNN-8-1	−0.52	−0.66	0.61	0.57	0.05	0.29	−0.25	−0.09	−0.05	−0.29	0.25	0.09	4.39	0.44
	qlNN-8-2	0.16	−0.15	−0.89	0.88	0.39	0.08	−0.10	−0.37	−0.39	−0.08	0.10	0.37	4.65	0.80
	qlNN-9-1	0.36	−0.74	0.10	0.29	−0.44	0.18	0.21	0.06	0.44	−0.18	−0.21	−0.06	3.04	0.80
	qlNN-12-1	−0.92	0.78	−0.01	0.16	−0.20	0.24	−0.04	0.01	0.20	−0.24	0.04	−0.01	3.48	0.26
	qlNN-12-2	−0.30	−0.27	0.86	−0.29	−0.30	0.44	−0.12	−0.02	0.30	−0.44	0.12	0.02	2.87	0.83
	qlNN-13-1	0.73	−0.22	0.24	−0.76	−0.35	0.02	−0.23	0.56	0.35	−0.02	0.23	−0.56	2.12	0.96
	qlNN-18-1	0.96	−0.06	−0.31	−0.60	−1.03	0.18	0.35	0.50	1.03	−0.18	−0.35	−0.50	3.21	2.99
	Total	□	□	□	□	□	□	□	□	□	□	□	□	35.566	10.407

a *E1, Harbin in 2015; E2, Keshan in 2015; E3, Acheng in 2016; E4, Shuangcheng in 2016; E5, Harbin in 2016; D1, normal density (2.2 × 10^5^ plants/ha); D2, high density (3 × 10^5^ plants/ha)*.

b add1, add2, add3, add4, genetic effects from Kenfeng 14, Kenfeng 15, Heinong 48, and Kenfeng 19, respectively; add1^*^D1, add2^*^D1, add3^*^D1, add4^*^D1 represent genetic effect of Kenfeng 14, Kenfeng 15, Heinong 48, Kenfeng 19 under D1; and add1^*^D2, add2^*^D2, add3^*^D2, add4^*^D2 represent allelic additive effect of Kenfeng 14, Kenfeng 15, Heinong 48, Kenfeng 19 under D2.

c *PVE_A_ and PVE_AD_ represent phenotypic variation ratio explained by additive and additive × density interaction effect for each of the QTL, respectively*.

By comparison of PVE_A_ and PVE_AD_, 15 QTL (qlNN-20-1, qlNN-12-1, qlNN-3-1, qlNN-3-2, qlNN-13-1, qlNN-19-1, qlNN-8-2, qlNN-8-1, qlNN-6-4, qlNN-17-2, qlNN-9-3, qlNN-18-1, qlNN-9-1, qlNN-18-2, and qlNN-12-2) expressed stably in two densities. Of these, qlNN-3-2 in E1, qlNN-9-2 and qlNN-14-1 in E4, and qlNN-3-1, qlNN-6-4, qlNN-8-1, qlNN-8-2, qlNN-9-1, qlNN-12-1, qlNN-12-2, qlNN-13-1, and qlNN-18-1 in E5 showed consistency with PVE_A_ over 2%.

Twelve QTL (qlNN-8-3, qlNN-2-1, qlNN-11-1, qlNN-14-1, qlNN-1-2, qlNN-17-1, qlNN-1-3, qlNN-10-2, qlNN-6-3, qlNN-10-1, qlNN-18-3, and qlNN-1-1) showed larger inconformity in various densities. Among these, qlNN-1-2 and qlNN-11-1 in E1, and qlNN-10-2 in E3 were expressed differently in specific density with PVE_AD_ more than 2%. Three QTL (qlNN-5-1, qlNN-9-2, qlNN-6-2) responded differently to density change in various environments.

### QTL by GWAS

By GWAS analysis, QTL associated with MSNN were detected all over the genome on 18 chromosomes except chromosomes 16 and 17 (Figure 3). Thirty-four QTL were found in D1, 18 in D2, and 34 in RD, in which five were simultaneously found in D2 and RD (Figure 4). In other words, a total of 81 QTL were found, 47 of which could explain 10.1–38.38% phenotypic variation (Supplementary Table 2). From the different environments, 10, 26, 25, 9, and 10 QTL were identified specifically in E1, E2, E3, E4, and E5, respectively, and one QTL (*qnRDNN*-13-3) was repeatedly identified in E1 and E5 (Figure 5). Twenty-four stable QTL could be found with multiple methods or in the environments, 19 of which could explain phenotypic variation more than 10% (Table 5). *qnNN*-4-1 (*qnRDNN*-4-1) was identified in E4D2 and E4RD with PVE of 11.684–28.71%; *qnNN*-4-2 was identified in E5D1 with PVE of 15.1315–15.3806%; *qnRDNN*-5-3 was identified in E2RD with PVE of 8.3101–18.7664%; *qnNN-*6-2 was identified in E4D2 with PVE of 10.7297–17.4975%; *qnRDNN*-7-1 was identified in E5RD with PVE of 9.9725–24.8721%; *qnNN-*7-2 was identified in E2D1 with the PVE of 20.9359–26.3968%; *qnNN-*7-4 was identified in E2D2 with PVE of 12.1529–13.9883%; *qnNN-*9-1 was identified in E2D1 with PVE of 5.1691–10.674%; *qnRDNN*-9-2 was identified in E3RD with PVE of 11.9235–13.3229%; *qnNN-*10-2 was identified in E4D1 with PVE of 9.1925–10.6283%; *qnNN-*11-1 was identified in E5D1 with PVE of 26.5631–26.5932%; *qnNN-*12-1 was identified in E1D1 with PVE of 15.6919–22.8492%; *qnRDNN-*13-1 was identified in E2RD with PVE of 10.5682–20.7672%; *qnNN-*13-2 (*qnRDNN*-13-2) was identified in E3D2 and E3RD with PVE of 4.6444–16.7223%; *qnNN-*13-3 (*qnRDNN*-13-3) was identified in E1D2, E1RD, and E5RD with PVE of 14.1841–18.659%; *qnNN-*14-1 (*qnRDNN*-14-1) was identified in E1D2 and E1RD with PVE of 24.0645–38.3834%; *qnNN-*15-1 was identified in E4D1 with PVE of 13.4194–24.9554%; *qnNN-*18-3 was identified in E2D1 with PVE of 11.7346–20.1023%; and *qnNN-*19-2 was identified in E2D1 and could explain phenotypic variation of 4.3512–10.302%.

**Table 5:** Genome-wide association studies for main stem node number (MSNN) detected by multiple methods under different densities in different environments.

**QTL**	**Marker**	**Chromosome**	**Marker position (bp)**	**Treatment^a^**	**Method**	**Effect**	**LOD score**	**r^**2**^ (%)^b^**
***qnNN*** **-4-1** **(** ***qnRDNN*** **-4-1)**	AX-157404156	Chr04	9,753,769	E4D2	ISIS EM-BLASSO, FASTmrMLM	1.45 1.27	5.83 5.83	28.71 21.55
				E4RD	ISIS EM-BLASSO, mrMLM	0.90 1.28	3.12 3.43	11.68 21.65
***qnNN-*** **4-2**	AX-157124243	Chr04	10,279,678	E5D1	FASTmrMLM, pLARmEB	1.14 1.13	3.07 3.31	15.38 15.13
*qnNN*-5-3	AX-157344915	Chr05	33,299,938	E3D1	FASTmrMLM, pLARmEB	−0.87 −0.90	4.17 3.39	8.76 9.25
***qnRDNN*** **-5-3**	AX-157510718	Chr05	35,447,656	E2RD	pLARmEB, FASTmrMLM	−0.84 −0.53	7.67 4.35	18.77 8.31
*qnNN*-5-4	AX-157217038	Chr05	37,951,491	E3D2	FASTmrMLM, pLARmEB	−0.78 −0.78	4.26 4.26	9.10 9.10
***qnNN*** **-6-2**	AX-117468788	Chr06	7,332,663	E4D2	ISIS EM-BLASSO, FASTmrMLM	0.89 1.14	3.12 3.12	10.73 17.50
***qnRDNN*** **-7-1**	AX-157553491	Chr07	17,986,150	E5RD	ISIS EM-BLASSO, pLARmEB, FASTmrMLM	−0.97 −0.65 −0.73	5.71 3.13 5.33	24.87 9.97 13.88
***qnNN*** **-7-2**	AX-157107526	Chr07	38,273,310	E2D1	ISIS EM-BLASSO, FASTmrEMMA, pLARmEB, mrMLM, FASTmrMLM	−0.97 −1.84 −0.89 −0.86 −0.95	6.49 6.33 7.98 7.02 10.67	26.40 22.15 22.41 20.94 25.17
***qnNN*** **-7-4**	AX-157299646	Chr07	42,911,447	E2D2	pLARmEB, FASTmrMLM	0.69 0.64	5.97 3.25	13.99 12.15
*qnNN*-8-4	AX-157333638	Chr08	46,075,240	E3D1	pLARmEB,	−0.87 −0.71	3.30 3.04	9.49 6.33
***qnNN*** **-9-1**	AX-157536173	Chr09	18,807,588	E2D1	pLARmEB, FASTmrMLM FASTmrMLM	−0.44 −0.64 −0.52	3.15 5.26 4.93	5.17 10.67 7.01
***qnRDNN*** **-9-2**	AX-157088086	Chr09	38,577,050	E3RD	ISIS EM-BLASSO,	−0.93 −2.03	5.03 3.65	11.92 13.32
***qnNN*** **-10-2**	AX-157499787	Chr10	44,669,350	E4D1	ISIS EM-BLASSO, FASTmrEMMA pLARmEB	0.71 0.69	3.37 3.29	10.63 9.19
***qnNN*** **-11-1**	AX-157134381	Chr11	30,526,558	E5D1	pLARmEB, FASTmrMLM	1.51 1.51	5.76 5.71	26.56 26.59
***qnNN*** **-12-1**	AX-157131535	Chr12	11,077,832	E1D1	pLARmEB, FASTmrMLM	−1.12 −1.27	5.48 3.99	15.69 22.85
***qnRDNN*** **-13-1**	AX-157183655	Chr13	12,074,020	E2RD	mrMLM, FASTmrMLM	0.92 0.62	3.64 4.80	20.77 10.57
***qnNN*** **-13-2** **(** ***qnRDNN*** **-13-2)**	AX-157244239	Chr13	28,227,088	E3D2	ISIS EM-BLASSO, pLARmEB, FASTmrMLM	1.18 0.89 0.89	6.90 3.70 3.70	16.72 9.63 9.63
				E3RD	pLARmEB	0.64	3.45	4.64
***qnNN*** **-13-3** **(** ***qnRDNN*** **-13-3)**	AX-157484481	Chr13	29,074,011	E1D2	FASTmrMLM	1.57	3.41	18.66
				E1RD	FASTmrMLM,	1.56	3.68	18.66
				E5RD	ISIS EM-BLASSO	0.90	3.75	14.18
***qnNN*** **-14-1** **(** ***qnRDNN*** **-14-1)**	AX-117471784	Chr 14	1,451,268	E1D2	ISIS EM-BLASSO	1.54	4.30	24.08
				E1RD	ISIS EM-BLASSO mrMLM	1.53 1.97	4.11 4.60	24.06 38.38
***qnNN*** **-15-1**	AX-157344171	Chr15	1,729,416	E4D1	ISIS EM-BLASSO, pLARmEB mrMLM, FASTmrMLM	0.88 0.87 1.12	4.28 4.19 3.76	15.18 13.42 24.96
***qnNN*** **-18-3**	AX-157574089	Chr18	3,169,778	E2D1	FASTmrEMMA, pLARmEB, mrMLM, FASTmrMLM	0.85 −1.61 −0.85 −0.95	3.10 3.92 6.08 6.28	14.20 11.73 16.25 20.10
*qnNN*-19-1	AX-157482492	Chr19	1,106,691	E3D2	pLARmEB, FASTmrMLM	−0.58 0.63	3.79 3.23	7.57 5.44
*qnRDNN*-19-6	AX-157559564	Chr 19	39,447,145	E2RD	pLARmEB, FASTmrMLM	0.63 0.38	3.23 3.01	5.43 3.74
***qnNN*** **-19-2**	AX-157561662	Chr19	42,175,830	E2D1	pLARmEB, mrMLM, FASTmrMLM	0.50	3.65	7.08

a *E1, Harbin in 2015; E2, Keshan in 2015; E3, Acheng in 2016; E4, Shuangcheng in 2016; E5, Harbin in 2016; D1, normal density (2.2 × 10^5^ plants/ha); D2, high density (3 × 10^5^ plants/ha)*.

b *r^2^, proportion of total phenotypic variation explained by each QTL. Bold font indicates QTL with PVE > 10%*.

Among the whole QTL identified by GWAS, 24 were co-located in the interval of QTL detected by linkage analysis (Figure 3), 15 of which could explain phenotypic variation more than 10%: *qnRDNN*-5-3 in *qlNN*-5-1, *qnRDNN*-9-2 in *qlRDNN*-9-1, *qnNN*-10-2 (*qlRDNN*-10-1) in *qlNN*-10-2, *qnRDNN*-13-1, *qnNN*-13-2 (*qnRDNN-*13-2) and *qnNN*-13-3 (*qnRDNN-*13-3) in *qlNN*-13-1 (*qlRDNN*-13-1), *qnNN*-18-3 in *qlNN*-18-1, *qnNN*-1-1 in *qlNN*-1-1*, qnNN*-1-3 in *qlNN*-1-3, *qnNN*-4-3 in *qlRDNN*-4-1, *qnRDNN*-5-1 in *qlNN*-5-1, *qnRDNN*-9-1 in *qlNN*-9-1, *qnNN*-9-4 in *qlNN*-9-2 (*qlRDNN-*9-2), *qnNN*-13-4 in *qlNN*-13-1 (*qlRDNN-*13-1), and *qnNN*-18-2 in *qlNN*-18-1. These QTL also could be considered stable because of the detection by linkage analysis and GWAS.

### Candidate Gene Prediction

In this study, genes were screened based on the physical position of the five stable QTL (genome length <600 kb and PVE > 10%) and the 27 stable one (PVE > 10%) mentioned above. In total, 549 genes were found, among which 265 were highly expressed in the stem or shoot tip. Then these genes were used to conduct pathway analysis in the KEGG database (http://www.kegg.jp).

**Figure 6:**
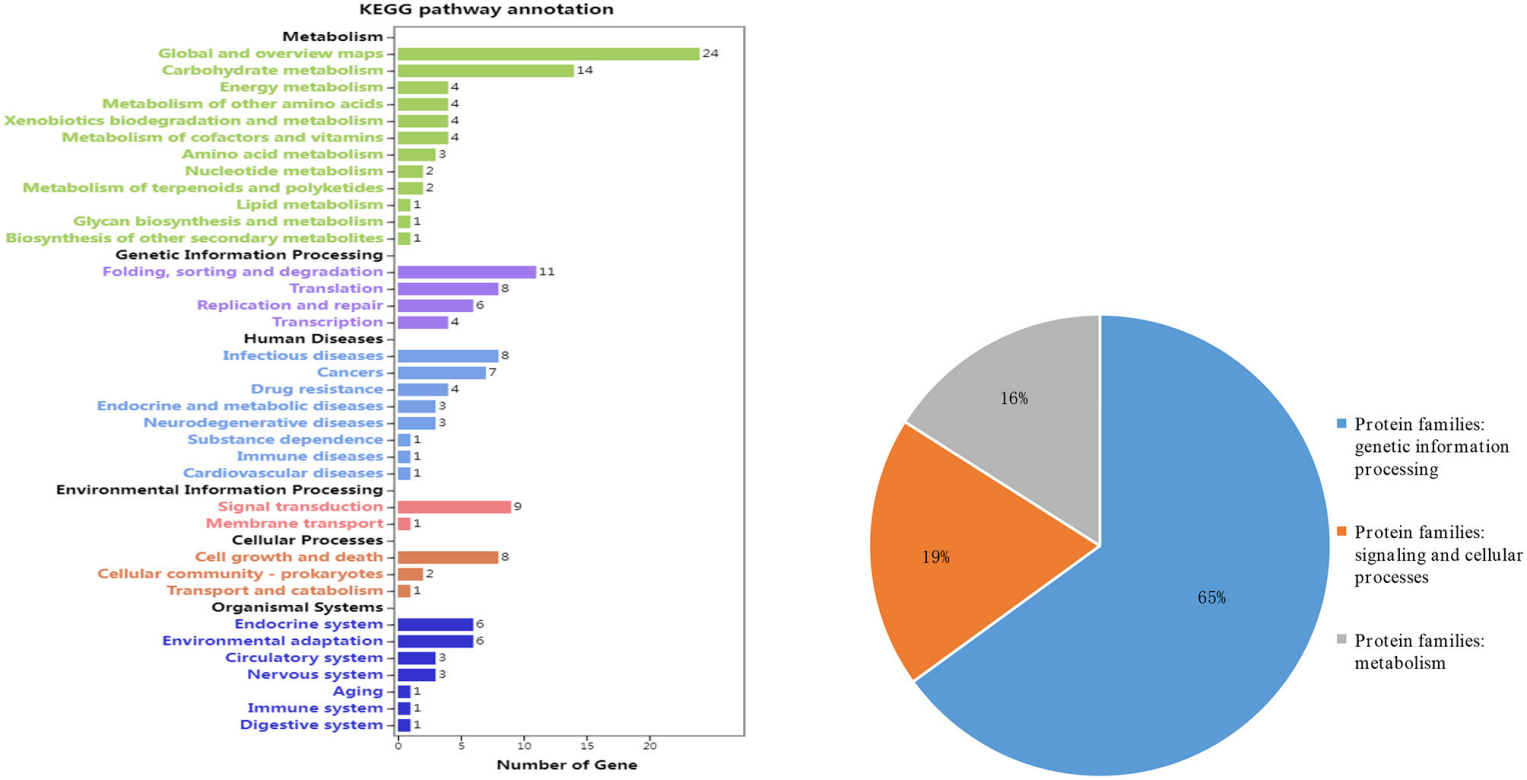
Information on pathways **(left)** and orthologous protein families **(right)** of main stem node number (MSNN)-annotated candidate genes.

A total of 106 genes (which accounted for 40%) were annotated and divided into 36 catalogs and three protein families (Figure 6). Among these genes, four (*Glyma.06G094400, Glyma.06G147600, Glyma.19G160800*, and *Glyma.19G161100*) were speculated as potential candidate genes to regulate MSNN (Table 6).

**Table 6:** Details of four potential candidate genes for main stem node number (MSNN).

**QTL**	**Gene ID**	**KEGG annotation**	**GO annotation**	**InterPro domains**	**Homologous gene**	**Biological process**
*qnNN*-6-2	Glyma.06G094400	YWHAE; 14-3-3 protein epsilon		IPR036815: 14-3-3 domain superfamily; IPR023410: 14-3-3 domain; IPR023409: 14-3-3 protein, conserved site; IPR000308: 14-3-3 protein	AT5G65430; AT5G10450	Response to cold; brassinosteroid mediated signaling pathway; positive regulation of protein catabolic process; response to cadmium ion; response to freezing
*qlNN*-6-2	Glyma.06G147600	BRI1; protein brassinosteroid insensitive 1 [EC: 2.7.10.1 2.7.11.1]	GO: 0006468: protein phosphorylation GO: 0016310: phosphorylation	IPR001245: Serine-threonine/tyrosine-protein kinase, catalytic domain IPR000719: Protein kinase domain IPR001611: Leucine-rich repeat IPR013210: Leucine-rich repeat-containing N-terminal, plant-type	AT3G13380; AT1G55610	Protein phosphorylation; phosphorylation; peptidyl-tyrosine phosphorylation
*qnNN*-19-2	Glyma.19G160800	trpA; tryptophan synthase alpha chain [EC: 4.2.1.20]	GO: 0006568: tryptophan metabolic process	IPR011060: Ribulose-phosphate binding barrel; IPR002028: Tryptophan synthase, alpha chain; IPR013785: Aldolase-type TIM barrel	AT4G02610	Tryptophan metabolic process; cellular amino acid biosynthetic process; auxin biosynthetic process
*qnNN*-19-2	Glyma.19G161100	IAA; auxin-responsive protein IAA	GO: 0009734: auxin-activated signaling pathway	IPR033389: AUX/IAA domain	AT4G14550; AT3G23050; AT3G04730; AT1G04250	Response to auxin; auxin-activated signaling pathway; lateral root morphogenesis

## Discussion

### Superiority of Using FW-RIL Population

In this study, a FW-RIL population was used for mapping, a simple mode of MAGIC (Kover et al., 2009) with four parents. It kept the advantage of the MAGIC population in abundant genetic variation. When mapping QTL, it could be applied to analyze allelic additive effects value from four parents. As long as there were differences between any two parents, QTL could be detected. For example, *qlNN*-1-1, *qlNN*-8-2, and *qlNN*-11-1 could not be detected in a bi-parent population derived from Kenfeng 14 × Kenfeng 15, because the allelic additive effects from the parents were approximately equivalent (Supplementary Table 1); so, the more allelic additive effect differences in the FW-RIL population, the greater the improvement made in QTL detection. Moreover, FW-RIL was an artificial population without the population structure problem, which was suitable for GWAS analysis.

Although the population size was relatively small, the combination of the linkage and GWAS analyses could improve mapping power. Furthermore, the experiment was conducted in three environments, which could compensate for the shortage in lower power in single environments. In summary, the statistical methods and multiple environment design could increase QTL detection power.

### Combination of Genetic Linkage Analysis and GWAS Analysis

Both the genetic linkage and GWAS analyses were the main methods to identify genome regions related to quantitative traits. As mentioned above, research studies have already combined the two methods to conduct target trait location analysis. In soybean, the combination of the two methods was used in several traits, such as seed size and shape (Hu et al., 2013), seed protein and oil content (Zhang et al., 2019), number of pods (Song et al., 2020), and plant height (Fang et al., 2020). Similar to the MAGIC population, four parents carried multiple allelic genotypes in FW-RIL, so it could conduct linkage and association analysis (Zhang et al., 2018; Li et al., 2019, 2020, 2021; Liu et al., 2019; Qi et al., 2020; Song et al., 2020; Tian et al., 2020; Wang et al., 2021). First, the principles of linkage and association were different: the former associated an interval (region) with a target trait, and the latter associated a position (SNP) with a target trait. Second, the genotype data used in linkage and association analysis were different: the former was based on a small number of markers, and the latter depended on the large amount of markers over the whole genome, so the combination of linkage and GWAS could increase the identification of genomic regions associated with target traits in FW-RIL. In this research, the IM and inclusive composite interval mapping methods were used in the linkage analysis, and mrMLM, FASTmrMLM, FASTmrEMMA, pLARmEB, and ISIS EM-BLASSO were used in the GWASs. A total of 38 QTL were identified by linkage analysis and 81 QTL were identified by GWAS, and 24 QTL were co-located in the interval of the identified QTL. The results indicated that the difference of the two methods in statistical principles and the genetic basis could complement each other and facilitate the detection of QTL.

### Density Response of MSNN

Plant density is considered to be an important factor affecting soybean yield and yield components, such as MSNN. Ikeda et al. (1994) reported that soybean yield increased as density increased because of increase in total node number, especially branch node number. In this study, the MSNN of most of the lines increased as the density increased. Some lines showed the opposite response to density increase, indicating that the expression of gene for MSNN was probably affected by the change in density. By combining linkage and GWAS analyses, 55 QTL were identified in D1, and 33 QTL were identified in D2, respectively. Only five of them were identified in both of the densities, while the rest were detected in the single density. The results showed that the genetic basis of the QTL for MSNN was significantly different in the two densities, and for the genotype, environments, densities and their interaction were all at work. Inspired by the conditional genetic effects (Zhu, 1995) based on a net-effect analysis, the effect of MSNN response to density was estimated by the removal of other factors except planting density increment. In total, 48 QTL for MSNN response to density were identified when planting density increased from 2.2 × 10^5^ (D1) to 3 × 10^5^ plants/ha (D2), which is more valuable for molecular assistance selection (MAS) on MSNN in a specific planting density. Besides, in terms of the additive effects of all QTL for MSNN response to density, Heinong 48 and Kenfeng 14 were relatively suitable parents for increasing and decreasing MSNN in the MAS of soybean breeding, respectively.

### Comparison of QTL Identified in Various Genetic Backgrounds

There were 25 QTL identified by linkage and 11 identified by GWAS listed in Soybase (https://www.soybase.org/search/qtllist_by_symbol.php). Among the 119 QTL identified this study, 10 had genome intervals that overlapped with published QTL node number (Figure 3). *qlNN*-2-1 was identified on chromosome 2 in genome intervals of 29,959,409–41,608,316 bp, overlapping with Node number 4-1 (38,221,027–40,699,300 bp) (Liu et al., 2011). *qlNN*-5-1was identified on chromosome 5 in genome intervals of 22,088,622–41,360,809 bp, overlapping with Node number 3-1 (35,971,621–38,939,759 bp) (Chen et al., 2007). *qlNN*-6-2 was identified on chromosome 6 in genome intervals of 11,860,267–12,150,538 bp, overlapping with Node number 5-1 (10,251,126–12,336,492 bp) (Moongkanna et al., 2011). *qlNN*-13-1 (*qlRDNN*-13-1) was identified on chromosome 13 in genome intervals of 444,838–43,052,819 bp, overlapping with Node numbers 1-5, 1-6, 1-7, 1-8 (Gai et al., 2007), and 2-3 (Zhang et al., 2004). *qlNN*-17-2 was identified on chromosome 17 in genome intervals of 7,296,590−9,660,500 bp, overlapping with Node number 7-1 (5,788,551–9,576,644 bp) (Li et al., 2010). *qnNN*-5-4 (37,951,491 bp) and *qnRDNN*-5-4 (38,349,709 bp) were identified in chromosome 5 and fell in the interval of Node number 3-1 (35,971,621–38,939,759 bp) (Chen et al., 2007); *qnNN*-6-3 (19,386,897 bp) was identified on chromosome 6 and fell in the interval of Node number 2-2 (19,370,872-20,218,893 bp) (Zhang et al., 2004). *qnRDNN*-13-1 (12,074,020 bp) and *qnNN*-13-1 (14,139,382 bp) were identified on chromosome 13 and fell in the interval of Node number 1-5 (10,199,530–15,306,234 bp) (Gai et al., 2007). The rest of 33 QTL identified by linkage and 76 QTL identified by GWAS were newly discovered, among which 37 with PVE > 10% were repeatedly identified with multiple density, environments, or methods (Supplementary Tables 1, 2). Consequently, this study probably would provide a great number of available genome regions and some potential high-confident candidate genes for MSNN.

### Candidate Gene Related With MSNN

It is known that only few genes were directly related to MSNN in different crops. A novel *ricMT* gene was highly expressed in stem nodes (Yu et al., 1998). *ZmMADS3* was expressed in the stem nodes of maize, and the transgenic maize reduced the number of nodes (Heuer et al., 2001). *Dt1* controlled the number of nodes in soybean by regulating stem growth habit (Bernard, 1972). Therefore, it is of great significance to explore potential candidate genes for MSNN. In this study, four among 106 genes were predicted for MSNN.

Brassinosteroids are essential plant hormones with significant effect on cell proliferation and elongation. Glyma.06G147600 was annotated as protein brassinosteroid insensitive 1 (BRI1). It has been demonstrated that BRI1 is a receptor kinase that transduces steroid signals across the plasma membrane, which is likely to be the primary brassinosteroids (BR) receptor in *Arabidopsis* (Wang et al., 2001). Glyma.06G094400 was annotated as 14-3-3 protein epsilon. 14-3-3 proteins were highly conserved regulatory proteins, which interact with diverse target proteins in a sequence-specific and phosphorylation-dependent manner (Bridges and Moorhead, 2005). They have been proved to be involved in many processes of metabolism, hormone signaling introduction, cell division, and responses to abiotic and biotic stress in plants (Chen et al., 2006; Takahashi et al., 2007; Swatek et al., 2011). 14-3-3 proteins participate in BR signal transduction by regulating the subcellular localization and activity of both BZR1 and BZR2/BES1, which are the key transcription factor of BR signal transduction (Gampala et al., 2007). Chae et al. (2016) found that 14-3-3 proteins bound to BRI1, a kind of BR-receptor kinase, and phosphorylated in a BR-dependent manner, demonstrating that 14-3-3 proteins play an important role in the BR signaling of *A. thaliana*. Therefore, Glyma.06G094400 and Glyma.06G147600 could play an important role in MSNN because they probably would regulate stem growth through BR signaling pathway.

Glyma.19G160800 is annotated as tryptophan synthase alpha chain. Tryptophan synthase is an enzyme that catalyzes the final two steps in the biosynthesis of tryptophan, which could be converted to indole acetic acid (IAA) *via* the indole acetaldehyde or indole acetonitrile pathway. Glyma.19G161100 is annotated as auxin-responsive protein IAA. In other words, the two genes function in plant growth by IAA indirectly or directly. IAA is well-known for its strong effect on stimulating elongation in isolated stem segments (Yang et al., 1996), which has previously demonstrated that stem elongation strongly responded to exogenous IAA in light-grown pea (Murayama and Ueda, 1973; Yang et al., 1993). Recent research studies further showed that an auxin gradient was involved in cell proliferation in *Arabidopsis* and rice (Wang et al., 2018), and that auxin could convert to other forms to keep homeostasis to regulate soybean stem growth and development through various pathways (Jiang et al., 2020). Obviously, the two genes have a function in soybean stem growth and might have a certain relationship with MSNN. The four genes are all related with plant hormone signal transduction. It is necessary to verify the function of these genes in the future.

## Conclusion

In this study, by combining linkage analysis and GWAS analysis, a total of 119 QTL associated with MSNN were identified in the FW-RIL population. Among them, 24 were simultaneously identified by the two methods. On the basis of the five QTL repeatedly detected in D1 and D2 and the 36 QTL for MSNN response to density, it was implied that a specific molecular mechanism controlled the MSNN response with the increase in plant density. In addition, 109 QTL were newly found, and four candidate genes were predicted to be closely related to MSNN. These genes could be of great value for MAS of soybean breeding.

## Data Availability Statement

The original contributions presented in the study are included in the article/Supplementary Material, further inquiries can be directed to the corresponding authors.

## Author Contributions

HN and W-XL: conceptualization. HZ, MS, RR, TY, HL, YH, CL, and XS: investigation. HN: resources, data curation, supervision, and funding acquisition. PW: writing—original draft preparation. BH and HN: writing—review and editing. W-XL: project administration. All authors have read and agreed to the published version of the manuscript.

## Conflict of Interest

The authors declare that the research was conducted in the absence of any commercial or financial relationships that could be construed as a potential conflict of interest.

## Publisher's Note

All claims expressed in this article are solely those of the authors and do not necessarily represent those of their affiliated organizations, or those of the publisher, the editors and the reviewers. Any product that may be evaluated in this article, or claim that may be made by its manufacturer, is not guaranteed or endorsed by the publisher.
