# Supplementary material for: QTL for Main Stem Node Number and Its Response to Plant Densities in 144 Soybean FW-RILs
Source: Front Plant Sci. 2021 Aug 20;12:666796. doi: 10.3389/fpls.2021.666796 (PMC8417731; doi:10.3389/fpls.2021.666796)
Supplement: Supplementary file 1 [file Data_Sheet_1.docx]

**Table S1 QTL for NNMS detected under different densities in five environments**

| QTL | Chromosome | Marker position (bp) | | Interval length(bp) | Trait name | Methods | LOD score | PVE(%) | Add1 | Add2 | Add3 | Add4 | LeftCI | RightCI | QTL reported in previous studies |
| --- | --- | --- | --- | --- | --- | --- | --- | --- | --- | --- | --- | --- | --- | --- | --- |
| *qlNN*-1-1 | Chr01 | 2872700 | 4150293 | 1,277,593 | E5D2 | ICIM | 3.80 | 8.76 | 0.88 | 0.69 | 0.25 | -1.82 | 20.5 | 24.5 | Novel |
| *qlNN*-1-2 | Chr01 | 51550517 | 51752485 | 201,968 | E1D1 | ICIM | 5.37 | 7.16 | -1.23 | 1.19 | 0.98 | -0.94 | 94.5 | 99.5 | Novel |
| *qlNN*-1-3 | Chr01 | 44457217 | 45810605 | 1,353,388 | E2D1 | ICIM | 3.19 | 11.01 | -0.02 | 0.73 | 0.24 | -0.94 | 131.5 | 134.5 | Novel |
|  |  |  |  |  | E2D1 | IM | 3.19 | 11.01 | -0.02 | 0.73 | 0.24 | -0.94 | 131.5 | 134.5 |  |
| *qlNN*-2-1 | Chr02 | 29959409 | 41608316 | 11,648,907 | E1D1 | IM | 3.14 | 6.64 | -0.24 | 1.98 | -1.16 | -0.58 | 157.5 | 167.5 | Node number 4-1(Liu et al., 2011) |
| *qlRDNN*-2-1 | Chr02 | 13751038 | 29959409 | 16,208,371 | E5RD | IM | 3.53 | 5.84 | 0.61 | 1.17 | -1.38 | -0.40 | 160.5 | 173.5 | Novel |
| *qlRDNN*-3-1 | Chr03 | 40556853 | 40765554 | 208,701 | E2RD | IM | 3.57 | 7.57 | -0.51 | 0.57 | 0.57 | -0.63 | 27.5 | 31.5 | Novel |
|  |  |  |  |  | E2RD | ICIM | 4.60 | 12.07 | -0.50 | 0.52 | 0.64 | -0.66 | 28.5 | 31.5 |  |
| *qlRDNN*-3-2 | Chr03 | 41177717 | 41397901 | 220,184 | E5RD | ICIM | 3.81 | 9.55 | -0.52 | 1.16 | -0.95 | 0.31 | 34.5 | 37.5 | Novel |
|  |  |  |  |  | E5RD | IM | 3.30 | 3.58 | -0.45 | 1.17 | -1.01 | 0.29 | 31.5 | 42.5 |  |
| *qlNN*-3-1  (*qlRDNN*-3-3) | Chr03 | 43985812 | 44751018 | 765,206 | E5D1 | ICIM | 4.61 | 9.54 | 0.14 | -0.96 | 1.32 | -0.50 | 53.5 | 54.5 | Novel |
|  |  |  |  |  | E5RD | IM | 3.34 | 3.91 | -1.88 | 1.16 | -0.22 | 0.94 | 53.5 | 54.5 |  |
| *qlNN*-3-2 | Chr03 | 36761362 | 36919868 | 158,506 | E1D1 | ICIM | 3.67 | 4.79 | -1.04 | 1.11 | 0.67 | -0.74 | 127.5 | 131.5 | Novel |
| *qlRDNN*-4-1 | Chr04 | 10948200 | 46772115 | 35,823,915 | E5RD | IM | 3.58 | 5.95 | 1.87 | -0.78 | -1.05 | -0.03 | 103.5 | 112.5 | Novel |
| *qlNN*-5-1 | Chr05 | 22088622 | 41360809 | 19,272,187 | E4D1 | IM | 3.05 | 6.16 | 0.25 | -1.01 | 0.65 | 0.12 | 261.5 | 278.5 | Node number 3-1 (Chen et al., 2007) |
| *qlNN*-6-1  (*qlRDNN*-6-1) | Chr06 | 49792442 | 49930570 | 138,128 | E1D2 | ICIM | 3.28 | 12.50 | 1.52 | -1.19 | -0.56 | 0.23 | 54.5 | 65.5 | Novel |
|  |  |  |  |  | E1D1 | ICIM | 4.87 | 5.74 | 1.72 | -0.41 | -1.41 | 0.10 | 65.5 | 72.5 |  |
|  |  |  |  |  | E1D2 | IM | 3.41 | 11.72 | 1.69 | -1.20 | -0.62 | 0.12 | 65.5 | 71.5 |  |
|  |  |  |  |  | E1RD | ICIM | 3.41 | 11.11 | 1.59 | -1.22 | -0.49 | 0.12 | 65.5 | 71.5 |  |
|  |  |  |  |  | E1RD | IM | 3.16 | 11.11 | 1.70 | -1.10 | -0.67 | 0.08 | 65.5 | 72.5 |  |
|  |  |  |  |  | E1D1 | IM | 3.37 | 5.40 | 2.56 | -0.77 | -1.42 | -0.37 | 64.5 | 73.5 |  |
| *qlNN*-6-2 | Chr06 | 11860267 | 12150538 | 290,271 | E4D2 | ICIM | 3.70 | 11.68 | 0.79 | 0.67 | -0.08 | -1.37 | 129.5 | 134.5 | Node number 5-1(Moongkanna et al., 2011) |
|  |  |  |  |  | E4D2 | IM | 3.70 | 8.82 | 0.79 | 0.67 | -0.08 | -1.37 | 129.5 | 134.5 |  |
|  |  |  |  |  | E3D1 | ICIM | 3.35 | 6.85 | 0.65 | 0.93 | 0.06 | -1.64 | 140.5 | 144.5 |  |
|  |  |  |  |  | E4D1 | ICIM | 4.04 | 8.55 | -0.23 | 0.77 | 0.41 | -0.96 | 140.5 | 144.5 |  |
|  |  |  |  |  | E3D1 | IM | 3.35 | 6.85 | 0.65 | 0.93 | 0.06 | -1.64 | 140.5 | 144.5 |  |
|  |  |  |  |  | E4D2 | IM | 3.80 | 9.13 | 0.31 | 0.86 | 0.50 | -1.67 | 139.5 | 144.5 |  |
| *qlNN*-6-3 | Chr06 | 9071096 | 10028802 | 957,706 | E4D2 | IM | 3.09 | 7.45 | -0.02 | 1.26 | -0.78 | -0.46 | 173.5 | 184.5 | Novel |
| *qlNN*-6-4 | Chr06 | 7513077 | 8324902 | 811,825 | E5D2 | IM | 3.57 | 12.41 | -0.24 | 1.96 | -0.67 | -1.05 | 193.5 | 197.5 | Novel |
| *qlNN*-8-1 | Chr08 | 11150960 | 11762527 | 611,567 | E5D2 | ICIM | 7.58 | 12.67 | -0.81 | -1.82 | 1.63 | 1.00 | 100.5 | 106.5 | Novel |
| *qlNN*-8-2 | Chr08 | 12471405 | 15750097 | 3,278,692 | E5D2 | ICIM | 4.40 | 7.14 | 1.37 | 0.60 | -0.73 | -1.24 | 116.5 | 121.5 | Novel |
|  |  |  |  |  | E5D1 | ICIM | 5.49 | 10.92 | 0.96 | 0.98 | -1.39 | -0.55 | 120.5 | 128.5 |  |
|  |  |  |  |  | E5D1 | IM | 4.72 | 11.56 | 0.88 | 1.23 | -1.44 | -0.67 | 119.5 | 128.5 |  |
|  |  |  |  |  | E3D1 | ICIM | 3.08 | 6.82 | 0.13 | 1.49 | -0.52 | -1.10 | 121.5 | 129.5 |  |
|  |  |  |  |  | E3D1 | IM | 3.08 | 6.82 | 0.13 | 1.49 | -0.52 | -1.10 | 121.5 | 129.5 |  |
| *qlNN*-8-3 | Chr08 | 1262329 | 43939372 | 42,677,043 | E3D2 | ICIM | 3.72 | 12.62 | -1.29 | -0.75 | 1.66 | 0.38 | 197.5 | 200.5 | Novel |
| *qlNN*-9-1 | Chr09 | 1206616 | 32032649 | 30,826,033 | E5D1 | IM | 3.74 | 13.47 | -0.13 | 0.22 | 1.68 | -1.77 | 0 | 11.5 | Novel |
| *qlRDNN*-9-1 | Chr09 | 38322082 | 38450352 | 128,270 | E5RD | ICIM | 3.28 | 7.50 | -0.44 | 1.13 | -0.66 | -0.02 | 53.5 | 70.5 | Novel |
| *qlNN*-9-2  (*qlRDNN*-9-2) | Chr09 | 38854540 | 39771599 | 917,059 | E1D2 | ICIM | 3.02 | 10.50 | -1.09 | -0.09 | -0.02 | 1.19 | 71.5 | 84.5 | Novel |
|  |  |  |  |  | E3D2 | ICIM | 3.07 | 7.94 | -0.91 | 0.97 | -0.86 | 0.81 | 85.5 | 91.5 |  |
|  |  |  |  |  | E2D2 | IM | 3.61 | 9.91 | -0.79 | 1.06 | 0.07 | -0.34 | 88.5 | 96.5 |  |
|  |  |  |  |  | E2RD | IM | 3.17 | 6.63 | -0.76 | 0.84 | 0.12 | -0.20 | 88.5 | 97.5 |  |
| *qlNN*-9-3 | Chr09 | 10662787 | 30675431 | 20,012,644 | E4D1 | IM | 3.29 | 5.81 | 0.28 | -0.04 | 0.64 | -0.88 | 131.5 | 134.5 | Novel |
|  |  |  |  |  | E4D2 | ICIM | 3.73 | 11.75 | -1.92 | 0.49 | 1.03 | 0.39 | 172.5 | 177.5 |  |
| *qlNN*-10-1 | Chr10 | 25651766 | 32225908 | 6,574,142 | E1D1 | IM | 3.63 | 7.19 | -0.68 | 1.82 | -1.67 | 0.52 | 19.5 | 33.5 | Novel |
| *qlNN*-10-2  (*qlRDNN*-10-1) | Chr10 | 44278379 | 45076309 | 797,930 | E3D1 | ICIM | 3.02 | 6.12 | -0.06 | 0.80 | -1.31 | 0.56 | 54.5 | 66.5 | Novel |
|  |  |  |  |  | E3D1 | IM | 3.02 | 6.12 | -0.06 | 0.80 | -1.31 | 0.56 | 54.5 | 66.5 |  |
|  |  |  |  |  | E2RD | IM | 3.37 | 8.41 | -1.05 | -0.16 | 0.56 | 0.65 | 84.5 | 96.5 |  |
|  |  |  |  |  | E2RD | ICIM | 3.72 | 11.02 | -0.75 | -0.43 | 0.59 | 0.60 | 87.5 | 95.5 |  |
| *qlNN*-11-1 | Chr11 | 10492106 | 13537525 | 3,045,419 | E1D1 | ICIM | 7.47 | 10.00 | 1.50 | 1.10 | -1.22 | -1.39 | 109.5 | 118.5 | Novel |
| *qlNN*-12-1 | Chr12 | 36350871 | 36550507 | 199,636 | E5D1 | ICIM | 3.34 | 6.05 | -1.46 | -0.05 | 0.39 | 1.13 | 60.5 | 66.5 | Novel |
| *qlNN*-12-2 | Chr12 | 2151310 | 2257434 | 106,124 | E5D2 | ICIM | 3.51 | 4.92 | 0.04 | -1.34 | 1.17 | 0.14 | 129.5 | 137.5 | Novel |
| *qlNN*-13-1  (*qlRDNN*-13-1) | Chr13 | 444838 | 43052819 | 42,607,981 | E4RD | ICIM | 3.26 | 10.07 | 2.29 | -0.62 | -0.90 | -0.77 | 59.5 | 66.5 | Node number 1-5, 1-6,1-7,1-8 (Gai et al.2007) ;Node number 2-3 (Zhang et al., 2004) |
|  |  |  |  |  | E4RD | IM | 3.26 | 10.07 | 2.29 | -0.62 | -0.90 | -0.77 | 59.5 | 66.5 |  |
|  |  |  |  |  | E5D2 | ICIM | 3.35 | 5.16 | 1.34 | 0.25 | 0.33 | -1.92 | 94.5 | 96.5 |  |
|  |  |  |  |  | E5D2 | IM | 3.05 | 8.31 | 1.81 | 0.07 | 0.05 | -1.93 | 94.5 | 96.5 |  |
| *qlNN*-14-1 | Chr14 | 45455320 | 45551130 | 95,810 | E4D1 | IM | 3.28 | 5.71 | -0.76 | 1.12 | -0.19 | -0.17 | 189.5 | 198.5 | Novel |
| *qlRDNN*-16-1 | Chr16 | 33455110 | 33660848 | 205,738 | E3RD | IM | 3.06 | 5.03 | -1.76 | 0.97 | 0.54 | 0.25 | 30.5 | 34.5 | Novel |
| *qlRDNN*-16-2 | Chr16 | 36571848 | 36656555 | 84,707 | E3RD | IM | 3.22 | 5.29 | -1.48 | 1.43 | 0.32 | -0.28 | 35.5 | 43.5 | Novel |
| *qlNN*-17-1  (*qlRDNN*-17-1) | Chr17 | 32054668 | 32261428 | 206,760 | E1D1 | ICIM | 9.51 | 12.96 | 0.82 | 1.95 | -1.20 | -1.57 | 68.5 | 69.5 | Novel |
|  |  |  |  |  | E1D1 | IM | 3.12 | 4.28 | -0.13 | 1.51 | -0.36 | -1.02 | 67.5 | 73.5 |  |
|  |  |  |  |  | E5RD | ICIM | 3.63 | 9.47 | -0.88 | 1.04 | -0.59 | 0.44 | 118.5 | 120.5 |  |
|  |  |  |  |  | E5RD | IM | 3.06 | 3.44 | -0.87 | 1.03 | -0.62 | 0.46 | 115.5 | 120.5 |  |
| *qlNN*-17-2 | Chr17 | 7296590 | 9660500 | 2,363,910 | E1D1 | ICIM | 6.19 | 8.16 | -1.09 | -1.22 | 1.15 | 1.16 | 148.5 | 151.5 | Node number 7-1 (Li et al., 2009) |
| *qlNN*-18-1 | Chr18 | 2759157 | 10333228 | 7,574,071 | E5D1 | ICIM | 4.89 | 10.02 | 1.09 | -1.32 | -0.67 | 0.90 | 30.5 | 33.5 | Novel |
|  |  |  |  |  | E5D2 | IM | 3.57 | 9.51 | 2.16 | -1.21 | -1.08 | 0.13 | 30.5 | 34.5 |  |
|  |  |  |  |  | E5D2 | ICIM | 3.45 | 6.72 | 2.10 | -1.33 | -0.65 | -0.12 | 30.5 | 36.5 |  |
| *qlNN*-18-2 | Chr18 | 1952392 | 15439539 | 13,487,147 | E1D1 | IM | 3.24 | 5.59 | -0.43 | -0.20 | -1.37 | 1.99 | 102.5 | 115.5 | Novel |
| *qlNN*-18-3 | Chr18 | 8077026 | 44401750 | 36,324,724 | E4D2 | ICIM | 3.06 | 9.12 | -1.21 | -0.07 | 0.77 | 0.51 | 163.5 | 173 | Novel |
| *qlNN*-19-1 | Chr19 | 30323974 | 30592702 | 268,728 | E4D1 | ICIM | 4.83 | 14.93 | -0.19 | 0.70 | -1.18 | 0.67 | 31.5 | 36.5 | Novel |
|  |  |  |  |  | E4D1 | IM | 4.34 | 10.73 | -0.03 | 0.68 | -1.26 | 0.61 | 30.5 | 37.5 |  |
| *qlNN*-20-1  (*qlRDNN*-20-1) | Chr20 | 11858130 | 35970092 | 24,111,962 | E2D2 | IM | 3.34 | 10.99 | -1.05 | -0.06 | 0.09 | 1.02 | 21.5 | 35.5 | Novel |
|  |  |  |  |  | E2RD | IM | 3.70 | 8.02 | -0.88 | -0.04 | 0.00 | 0.92 | 24.5 | 36.5 |  |

E1:Harbin in 2015; E2:Keshan in 2015; E3:Acheng in 2016; E4:Shuangcheng in 2016; E5:Harbin in 2016.

D1:the first (normal)density (2.2×10^5^ plant/ha) ; D2:the second(high)density(3.0×10^5^ plant/ha); RD: response to density.

PVE:phenotypic variation explanation ratio.

Add1,Add2,Add3,Add4:additive effects from Kenfeng14, Kenfeng 15, Heinong 48, Kenfeng 19.

**Table S2 QTN for NNMS detected under different densities in five environments**

| QTN |  | Chromosome | Marker position (bp) | Trait name^a^ | Method | QTN effect | LOD score | lgP | r^2^ (%)^b^ | MAF^c^ | QTN reported in previous studies |
| --- | --- | --- | --- | --- | --- | --- | --- | --- | --- | --- | --- |
| *qnNN*-1-1 | AX-117066440 | Chr01 | 4013272 | E5D2 | ISIS EM-BLASSO | -1.03 | 4.43 | 5.20 | 14.24 | 0.31 | Novel |
| *qnNN*-1-2 | AX-157177877 | Chr01 | 34330582 | E5D1 | ISIS EM-BLASSO | -0.91 | 3.73 | 4.47 | 9.78 | 0.37 | Novel |
| *qnNN*-1-3 | AX-157531462 | Chr01 | 45032976 | E3D2 | ISIS EM-BLASSO | -0.82 | 4.91 | 5.70 | 10.10 | 0.48 | Novel |
| *qnNN*-2-1 | AX-157519151 | Chr02 | 30180558 | E3D2 | ISIS EM-BLASSO | 0.89 | 4.35 | 5.12 | 9.09 | 0.19 | Novel |
| *qnRDNN*-2-1 | AX-157558979 | Chr02 | 47315525 | E4RD | ISIS EM-BLASSO | 1.12 | 3.40 | 4.12 | 11.30 | 0.17 | Novel |
| *qnRDNN*-3-1 | AX-157262862 | Chr03 | 3627592 | E3RD | pLARmEB | -0.72 | 4.82 | 5.61 | 7.29 | 0.49 | Novel |
| *qnRDNN*-3-2 | AX-157459508 | Chr03 | 31790731 | E2RD | pLARmEB | -0.32 | 3.14 | 3.84 | 2.64 | 0.32 | Novel |
| *qnRDNN*-3-3 | AX-157098940 | Chr03 | 33991515 | E3RD | ISIS EM-BLASSO | 0.71 | 4.48 | 5.25 | 5.71 | 0.28 | Novel |
| *qnN*N-3-1 | AX-157495612 | Chr03 | 36922430 | E3D2 | ISIS EM-BLASSO | 0.77 | 4.13 | 4.89 | 5.67 | 0.17 | Novel |
| *qnRDNN*-3-4 | AX-157305932 | Chr03 | 39028294 | E3RD | FASTmrMLM | 0.71 | 3.09 | 3.80 | 7.00 | 0.38 | Novel |
| *qnNN*-4-1  (*qnRDNN*-4-1) | AX-157404156 | Chr04 | 9753769 | E4D2 | FASTmrMLM | 1.27 | 5.83 | 6.66 | 21.55 | 0.39 | Novel |
|  |  |  |  | E4D2 | ISIS EM-BLASSO | 1.45 | 5.83 | 6.66 | 28.71 | 0.39 |  |
|  |  |  |  | E4RD | mrMLM | 1.28 | 3.43 | 4.15 | 21.65 | 0.39 |  |
|  |  |  |  | E4RD | ISIS EM-BLASSO | 0.90 | 3.12 | 3.82 | 11.68 | 0.39 |  |
| *qnNN*-4-2 | AX-157124243 | Chr04 | 10279678 | E5D1 | FASTmrMLM | 1.14 | 3.07 | 3.77 | 15.38 | 0.41 | Novel |
|  |  |  |  | E5D1 | pLARmEB | 1.13 | 3.31 | 4.02 | 15.13 | 0.41 |  |
| *qnNN*-4-3 | AX-157440961 | Chr04 | 44895626 | E5D2 | ISIS EM-BLASSO | 1.19 | 4.29 | 5.06 | 22.48 | 0.50 | Novel |
| *qnNN*-4-4 | AX-157109855 | Chr04 | 48477117 | E2D1 | FASTmrMLM | 0.54 | 4.80 | 5.59 | 7.91 | 0.42 | Novel |
| *qnNN*-5-1 | AX-157322573 | Chr05 | 2165025 | E2D2 | pLARmEB | -0.70 | 3.33 | 4.05 | 13.98 | 0.42 | Novel |
| *qnNN*-5-2 | AX-117050246 | Chr05 | 10161902 | E1D1 | FASTmrMLM | -1.05 | 4.31 | 5.08 | 16.08 | 0.40 | Novel |
| *qnRDNN*-5-1 | AX-157477303 | Chr05 | 33188385 | E4RD | mrMLM | -1.11 | 3.23 | 3.94 | 16.87 | 0.43 | Novel |
| *qnNN*-5-3 | AX-157344915 | Chr05 | 33299938 | E3D1 | FASTmrMLM | -0.87 | 4.17 | 4.93 | 8.76 | 0.34 | Novel |
|  |  |  |  | E3D1 | pLARmEB | -0.90 | 3.39 | 4.11 | 9.25 | 0.34 |  |
| *qnRDNN*-5-2 | AX-116913150 | Chr05 | 33988564 | E2RD | ISIS EM-BLASSO | -0.46 | 3.35 | 4.06 | 6.88 | 0.48 | Novel |
| *qnRDNN*-5-3 | AX-157510718 | Chr05 | 35447656 | E2RD | FASTmrMLM | -0.53 | 4.35 | 5.11 | 8.31 | 0.34 | Novel |
|  |  |  |  | E2RD | pLARmEB | -0.84 | 7.67 | 8.55 | 18.77 | 0.34 |  |
| *qnNN*-5-4 | AX-157217038 | Chr05 | 37951491 | E3D2 | FASTmrMLM | -0.78 | 4.26 | 5.02 | 9.10 | 0.48 | Node number 3-1(Chen et al., 2007) |
|  |  |  |  | E3D2 | pLARmEB | -0.78 | 4.26 | 5.02 | 9.10 | 0.48 |  |
| *qnRDNN*-5-4 | AX-116935292 | Chr05 | 38349709 | E3RD | pLARmEB | -0.47 | 3.12 | 3.82 | 3.07 | 0.49 | Novel |
| *qnNN*-6-1 | AX-157130866 | Chr06 | 3212313 | E3D1 | ISIS EM-BLASSO | -1.32 | 3.33 | 4.05 | 13.82 | 0.19 | Novel |
| *qnNN*-6-2 | AX-117468788 | Chr06 | 7332663 | E4D2 | FASTmrMLM | 1.14 | 3.12 | 3.83 | 17.50 | 0.42 | Novel |
|  |  |  |  | E4D2 | ISIS EM-BLASSO | 0.89 | 3.12 | 3.83 | 10.73 | 0.42 |  |
| *qnRDNN*-6-1 | AX-157417138 | Chr06 | 8456097 | E3RD | ISIS EM-BLASSO | -0.93 | 5.39 | 6.20 | 11.66 | 0.42 | Novel |
| *qnRDNN*-6-2 | AX-157289265 | Chr06 | 9091101 | E5RD | pLARmEB | 0.86 | 3.17 | 3.88 | 9.76 | 0.13 | Novel |
| *qnNN*-6-3 | AX-157076868 | Chr06 | 19386897 | E2D1 | FASTmrMLM | 0.61 | 4.61 | 5.39 | 8.06 | 0.23 | Node number 2-2 (Zhang et al., 2004) |
| *qnRDNN*-6-3 | AX-157082832 | Chr06 | 50683002 | E2RD | ISIS EM-BLASSO | 0.51 | 4.15 | 4.91 | 8.47 | 0.42 | Novel |
| *qnNN*-6-4 | AX-157318854 | Chr06 | 50968151 | E3D1 | pLARmEB | 0.91 | 3.45 | 4.17 | 10.45 | 0.45 | Novel |
| *qnNN*-7-1 | AX-157521602 | Chr07 | 2827321 | E5D1 | ISIS EM-BLASSO | -1.12 | 4.75 | 5.53 | 14.78 | 0.39 | Novel |
| *qnRDNN*-7-1 | AX-157553491 | Chr07 | 17986150 | E5RD | FASTmrMLM | -0.73 | 5.33 | 6.14 | 13.88 | 0.44 | Novel |
|  |  |  |  | E5RD | pLARmEB | -0.65 | 3.13 | 3.84 | 9.97 | 0.44 |  |
|  |  |  |  | E5RD | ISIS EM-BLASSO | -0.97 | 5.71 | 6.53 | 24.87 | 0.44 |  |
| *qnNN*-7-2 | AX-157107526 | Chr07 | 38273310 | E2D1 | mrMLM | -0.86 | 7.02 | 7.89 | 20.94 | 0.46 | Novel |
|  |  |  |  | E2D1 | FASTmrMLM | -0.95 | 10.67 | 11.62 | 25.17 | 0.44 |  |
|  |  |  |  | E2D1 | FASTmrEMMA | -1.84 | 6.33 | 7.18 | 22.15 | 0.44 |  |
|  |  |  |  | E2D1 | pLARmEB | -0.89 | 7.98 | 8.87 | 22.41 | 0.44 |  |
|  |  |  |  | E2D1 | ISIS EM-BLASSO | -0.97 | 6.49 | 7.34 | 26.40 | 0.44 |  |
| *qnNN*-7-3 | AX-157259315 | Chr07 | 38426488 | E3D1 | FASTmrMLM | -0.75 | 3.75 | 4.49 | 7.02 | 0.47 | Novel |
| *qnNN*-7-4 | AX-157299646 | Chr07 | 42911447 | E2D2 | FASTmrMLM | 0.64 | 3.25 | 3.96 | 12.15 | 0.41 | Novel |
|  |  |  |  | E2D2 | pLARmEB | 0.69 | 5.97 | 6.80 | 13.99 | 0.41 |  |
| *qnNN*-8-1 | AX-157348715 | Chr08 | 2011324 | E4D1 | ISIS EM-BLASSO | 0.58 | 3.31 | 4.02 | 9.83 | 0.46 | Novel |
| *qnNN*-8-2 | AX-157423718 | Chr08 | 2012335 | E4D1 | pLARmEB | 0.59 | 3.27 | 3.98 | 9.05 | 0.45 | Novel |
| *qnNN*-8-3 | AX-117054441 | Chr08 | 6807597 | E1D1 | pLARmEB | -0.90 | 3.59 | 4.33 | 6.32 | 0.18 | Novel |
| *qnNN*-8-4 | AX-157333638 | Chr08 | 46075240 | E3D1 | FASTmrMLM | -0.71 | 3.04 | 3.74 | 6.33 | 0.49 | Novel |
|  |  |  |  | E3D1 | pLARmEB | -0.87 | 3.30 | 4.01 | 9.49 | 0.49 |  |
| *qnRDNN*-8-1 | AX-117055803 | Chr08 | 46278339 | E4RD | mrMLM | -1.62 | 4.14 | 4.90 | 19.31 | 0.17 | Novel |
| *qnRDNN*-9-1 | AX-157338403 | Chr09 | 6739360 | E5RD | pLARmEB | 0.86 | 3.99 | 4.75 | 13.67 | 0.23 | Novel |
| *qnNN*-9-1 | AX-157536173 | Chr09 | 18807588 | E2D1 | mrMLM | -0.64 | 5.26 | 6.06 | 10.67 | 0.38 | Novel |
|  |  |  |  | E2D1 | FASTmrMLM | -0.52 | 4.93 | 5.73 | 7.01 | 0.36 |  |
|  |  |  |  | E2D1 | pLARmEB | -0.44 | 3.15 | 3.85 | 5.17 | 0.36 |  |
| *qnNN*-9-2 | AX-116951880 | Chr09 | 30791595 | E2D1 | ISIS EM-BLASSO | 0.73 | 6.49 | 7.34 | 14.20 | 0.39 | Novel |
| *qnNN*-9-3 | AX-157350551 | Chr09 | 33975686 | E2D2 | FASTmrMLM | 0.54 | 3.02 | 3.71 | 8.05 | 0.34 | Novel |
| *qnRDNN*-9-2 | AX-157088086 | Chr09 | 38577050 | E3RD | FASTmrEMMA | -2.03 | 3.65 | 4.39 | 13.32 | 0.40 | Novel |
|  |  |  |  | E3RD | ISIS EM-BLASSO | -0.93 | 5.03 | 5.83 | 11.92 | 0.40 |  |
| *qnNN*-9-4 | AX-116921156 | Chr09 | 39488670 | E3D2 | ISIS EM-BLASSO | -1.34 | 6.72 | 7.57 | 17.10 | 0.21 | Novel |
| *qnNN*-10-1 | AX-157186709 | Chr10 | 10340153 | E3D1 | FASTmrMLM | -0.88 | 3.26 | 3.97 | 8.62 | 0.30 | Novel |
| *qnNN*-10-2 | AX-157499787 | Chr10 | 44669350 | E4D1 | pLARmEB | 0.69 | 3.29 | 4.00 | 9.19 | 0.20 | Novel |
|  |  |  |  | E4D1 | ISIS EM-BLASSO | 0.71 | 3.37 | 4.09 | 10.63 | 0.20 |  |
| *qnNN*-10-3 | AX-157413678 | Chr10 | 46587678 | E1D1 | pLARmEB | -1.18 | 4.42 | 5.19 | 10.66 | 0.14 | Novel |
| *qnNN*-11-1 | AX-157134381 | Chr11 | 30526558 | E5D1 | FASTmrMLM | 1.51 | 5.71 | 6.53 | 26.59 | 0.42 | Novel |
|  |  |  |  | E5D1 | pLARmEB | 1.51 | 5.76 | 6.58 | 26.56 | 0.42 |  |
| *qnNN*-12-1 | AX-157131535 | Chr12 | 11077832 | E1D1 | FASTmrMLM | -1.27 | 3.99 | 4.74 | 22.85 | 0.30 | Novel |
|  |  |  |  | E1D1 | pLARmEB | -1.12 | 5.48 | 6.29 | 15.69 | 0.30 |  |
| *qnNN*-12-2 | AX-157277277 | Chr12 | 12715775 | E1D1 | ISIS EM-BLASSO | -0.97 | 3.82 | 4.56 | 12.50 | 0.28 | Novel |
| *qnRDNN*-12-1 | AX-157089652 | Chr12 | 34469930 | E3RD | pLARmEB | -0.87 | 4.90 | 5.69 | 9.84 | 0.40 | Novel |
| *qnRDNN*-13-1 | AX-157183655 | Chr13 | 12074020 | E2RD | mrMLM | 0.92 | 3.64 | 4.37 | 20.77 | 0.31 | Node number 1-5(Gai et al., 2007) |
|  |  |  |  | E2RD | FASTmrMLM | 0.62 | 4.80 | 5.58 | 10.57 | 0.29 |  |
| *qnNN*-13-1 | AX-157251212 | Chr13 | 14139382 | E2D2 | pLARmEB | -0.54 | 3.89 | 4.64 | 8.75 | 0.48 | Node number 1-5(Gai et al., 2007) |
| *qnNN*-13-2  (*qnRDNN*-13-2) | AX-157244239 | Chr13 | 28227088 | E3D2 | FASTmrMLM | 0.89 | 3.70 | 4.44 | 9.63 | 0.30 | Novel |
|  |  |  |  | E3D2 | pLARmEB | 0.89 | 3.70 | 4.44 | 9.63 | 0.30 |  |
|  |  |  |  | E3D2 | ISIS EM-BLASSO | 1.18 | 6.90 | 7.76 | 16.72 | 0.30 |  |
|  |  |  |  | E3RD | pLARmEB | 0.64 | 3.45 | 4.18 | 4.64 | 0.30 |  |
| *qnNN*-13-3  (*qnRDNN*-13-3) | AX-157484481 | Chr13 | 29074011 | E1D2 | FASTmrMLM | 1.57 | 3.41 | 4.13 | 18.66 | 0.18 | Novel |
| *qnNN*-13-3 | AX-157484481 | Chr13 | 29074011 | E1RD | FASTmrMLM | 1.56 | 3.68 | 4.42 | 18.66 | 0.18 | Novel |
|  |  |  |  | E5RD | FASTmrMLM | 0.00 | 3.73 | 4.47 | 0.00 | 0.19 |  |
|  |  |  |  | E5RD | ISIS EM-BLASSO | 0.90 | 3.75 | 4.49 | 14.18 | 0.19 |  |
| *qnNN*-13-4 | AX-157341798 | Chr13 | 32986323 | E1D1 | ISIS EM-BLASSO | -1.14 | 4.78 | 5.57 | 11.54 | 0.16 | Novel |
| *qnNN*-14-1  (*qnRDNN*-14-1) | AX-117471784 | Chr14 | 1451268 | E1D2 | ISIS EM-BLASSO | 1.54 | 4.30 | 5.06 | 24.08 | 0.31 | Novel |
|  |  |  |  | E1RD | mrMLM | 1.97 | 4.60 | 5.38 | 38.38 | 0.30 |  |
|  |  |  |  | E1RD | ISIS EM-BLASSO | 1.53 | 4.11 | 4.87 | 24.06 | 0.31 |  |
| *qnNN*-14-2 | AX-157323950 | Chr14 | 7134081 | E1D1 | ISIS EM-BLASSO | -0.76 | 4.57 | 5.35 | 8.81 | 0.48 | Novel |
| *qnRDNN*-14-2 | AX-157088368 | Chr14 | 25007323 | E3RD | pLARmEB | -0.91 | 5.79 | 6.61 | 11.65 | 0.45 | Novel |
| *qnNN*-15-1 | AX-157344171 | Chr15 | 1729416 | E4D1 | mrMLM | 1.12 | 3.76 | 4.50 | 24.96 | 0.19 | Novel |
|  |  |  |  | E4D1 | FASTmrMLM | 0.85 | 3.10 | 3.80 | 14.20 | 0.21 |  |
|  |  |  |  | E4D1 | pLARmEB | 0.87 | 4.19 | 4.95 | 13.42 | 0.21 |  |
|  |  |  |  | E4D1 | ISIS EM-BLASSO | 0.88 | 4.28 | 5.04 | 15.18 | 0.21 |  |
| *qnRDNN*-15-1 | AX-157490165 | Chr15 | 4265524 | E2RD | ISIS EM-BLASSO | -0.37 | 3.48 | 4.21 | 4.58 | 0.48 | Novel |
| *qnNN*-15-2 | AX-157148563 | Chr15 | 43497078 | E1D1 | ISIS EM-BLASSO | 0.00 | 3.30 | 4.01 | 0.00 | 0.40 | Novel |
| *qnRDNN*-15-2 | AX-157063528 | Chr15 | 50377398 | E2RD | pLARmEB | 0.63 | 5.91 | 6.74 | 11.92 | 0.49 | Novel |
| *qnNN*-15-3 | AX-157527183 | Chr15 | 50491392 | E2D2 | pLARmEB | 0.57 | 4.66 | 5.44 | 9.59 | 0.49 | Novel |
| *qnNN*-18-1 | AX-157566375 | Chr18 | 1271382 | E1D1 | pLARmEB | -0.76 | 3.78 | 4.52 | 6.34 | 0.29 | Novel |
| *qnNN*-18-2 | AX-157137471 | Chr18 | 3098286 | E2D1 | ISIS EM-BLASSO | -0.84 | 3.35 | 4.07 | 14.97 | 0.25 | Novel |
| *qnNN*-18-3 | AX-157574089 | Chr18 | 3169778 | E2D1 | mrMLM | -0.95 | 6.28 | 7.13 | 20.10 | 0.24 | Novel |
|  |  |  |  | E2D1 | FASTmrMLM | -0.58 | 3.79 | 4.53 | 7.57 | 0.24 |  |
|  |  |  |  | E2D1 | FASTmrEMMA | -1.61 | 3.92 | 4.67 | 11.73 | 0.24 |  |
|  |  |  |  | E2D1 | pLARmEB | -0.85 | 6.08 | 6.91 | 16.25 | 0.24 |  |
| *qnNN*-18-4 | AX-157219531 | Chr18 | 51640237 | E3D1 | FASTmrMLM | 0.88 | 4.32 | 5.09 | 9.62 | 0.49 | Novel |
| *qnNN*-18-5 | AX-157592038 | Chr18 | 56694615 | E2D1 | mrMLM | 0.58 | 3.68 | 4.42 | 9.28 | 0.43 | Novel |
| *qnRDNN*-19-1 | AX-157099455 | Chr19 | 1069123 | E3RD | ISIS EM-BLASSO | 0.94 | 5.70 | 6.52 | 12.34 | 0.48 | Novel |
| *qnNN*-19-1  (*qnRDNN*-19-2) | AX-157482492 | Chr19 | 1106691 | E3D2 | FASTmrMLM | 0.63 | 3.23 | 3.93 | 5.43 | 0.41 | Novel |
|  |  |  |  | E3D2 | pLARmEB | 0.63 | 3.23 | 3.93 | 5.44 | 0.41 |  |
|  |  |  |  | E3RD | FASTmrMLM | 0.87 | 4.49 | 5.27 | 9.71 | 0.41 |  |
| *qnRDNN*-19-3 | AX-157429873 | Chr19 | 1201791 | E3RD | pLARmEB | 1.05 | 5.94 | 6.77 | 15.54 | 0.49 | Novel |
| *qnRDNN*-19-4 | AX-157232103 | Chr19 | 38285471 | E2RD | mrMLM | -1.32 | 5.01 | 5.80 | 30.38 | 0.18 | Novel |
| *qnRDNN*-19-5 | AX-157058029 | Chr19 | 38449056 | E2RD | FASTmrMLM | -0.73 | 4.17 | 4.93 | 13.31 | 0.22 | Novel |
| *qnRDNN*-19-6 | AX-157559564 | Chr19 | 39447145 | E2RD | FASTmrMLM | 0.50 | 3.65 | 4.39 | 7.08 | 0.30 | Novel |
|  |  |  |  | E2RD | pLARmEB | 0.38 | 3.01 | 3.71 | 3.74 | 0.30 |  |
| *qnRDNN*-19-7 | AX-157283594 | Chr19 | 40232802 | E2RD | ISIS EM-BLASSO | 0.76 | 5.42 | 6.23 | 15.32 | 0.26 | Novel |
| *qnRDNN*-19-8 | AX-157472925 | Chr19 | 40419433 | E2RD | pLARmEB | 0.65 | 3.10 | 3.80 | 10.59 | 0.28 | Novel |
| *qnNN*-19-2 | AX-157561662 | Chr19 | 42175830 | E2D1 | mrMLM | 0.62 | 3.49 | 4.21 | 10.30 | 0.44 | Novel |
|  |  |  |  | E2D1 | FASTmrMLM | 0.40 | 3.32 | 4.03 | 4.35 | 0.44 |  |
|  |  |  |  | E2D1 | pLARmEB | 0.50 | 3.69 | 4.43 | 6.67 | 0.44 |  |
| *qnRDNN*-19-9 | AX-157222566 | Chr19 | 44236837 | E3RD | ISIS EM-BLASSO | -1.01 | 4.02 | 4.77 | 8.53 | 0.16 | Novel |
| *qnNN*-20-1 | AX-157272196 | Chr20 | 42904720 | E5D1 | ISIS EM-BLASSO | 1.30 | 4.87 | 5.66 | 19.90 | 0.39 | Novel |

^a^E1:Harbin in 2015; E2:Keshan in 2015; E3:Acheng in 2016; E4:Shuangcheng in 2016; E5:Harbin in 2016.

D1:the first (normal)density (2.2×10^5^ plant/ha) ; D2:the second(high)density(3.0×10^5^ plant/ha); RD: response to density.

^b^r^2^:proportion of total phenotypic variation explained by each QTN.

^c^MAF: minor allele frequency.
